# Human cooperation with artificial agents varies across countries

**DOI:** 10.1038/s41598-025-92977-8

**Published:** 2025-03-22

**Authors:** Jurgis Karpus, Risako Shirai, Julia Tovar Verba, Rickmer Schulte, Maximilian Weigert, Bahador Bahrami, Katsumi Watanabe, Ophelia Deroy

**Affiliations:** 1 Faculty of Philosophy, Philosophy of Science and Religious Studies, LMU, Munich, Germany; 2 Faculty of Science and Engineering, Waseda University, Tokyo, Japan; 3 Japan Society for the Promotion of Science, Tokyo, Japan; 4 Division of Evolutionary Biology, Faculty of Biology, LMU, Munich, Germany; 5 Statistical Consulting Unit StaBLab, Department of Statistics, LMU, Munich, Germany; 6 Faculty of Psychology and Educational Sciences, LMU, Munich, Germany; 7 Munich Center for Neurosciences—Brain & Mind, Planegg-Martinsried, Germany; 8 Centre for Adaptive Rationality, Max Planck Institute for Human Development, Berlin, Germany; 9 Department of Psychology, Royal Holloway, University of London, Egham, UK; 10 Institute of Philosophy, School of Advanced Study, University of London, London, UK

**Keywords:** Human–AI interaction, Algorithm exploitation, Cross-cultural comparison, Cooperation, Psychology, Human behaviour

## Abstract

People are keen to exploit cooperative artificial agents for selfish gain. While this phenomenon has been observed in numerous Western societies, we show here that it is absent in Japan. We examined people’s willingness to cooperate with artificial agents and humans in two classic economic games requiring a choice between self interest and mutual benefit. Our participants in the United States cooperated with artificial agents significantly less than they did with humans, whereas participants in Japan exhibited equivalent levels of cooperation with both types of co-player. We found a notable difference in how people felt about exploiting their cooperative partner: people in Japan emotionally treated artificial agents and humans alike, whereas people in the United States felt bad about exploiting humans, but not machines. Our findings underscore the necessity for nuanced cultural considerations in the design and implementation of such technology across diverse societies

## Introduction

The growing popularity of chatGPT and the global competition to create the best new internet search engine show that new forms of technology can create both exciting opportunities and unexpected challenges. These experiences are not limited to any one culture or society, but are universal. However, the way people use technology and tackle novel challenges that are associated with it can differ from one place to another.

**Fig. 1:**
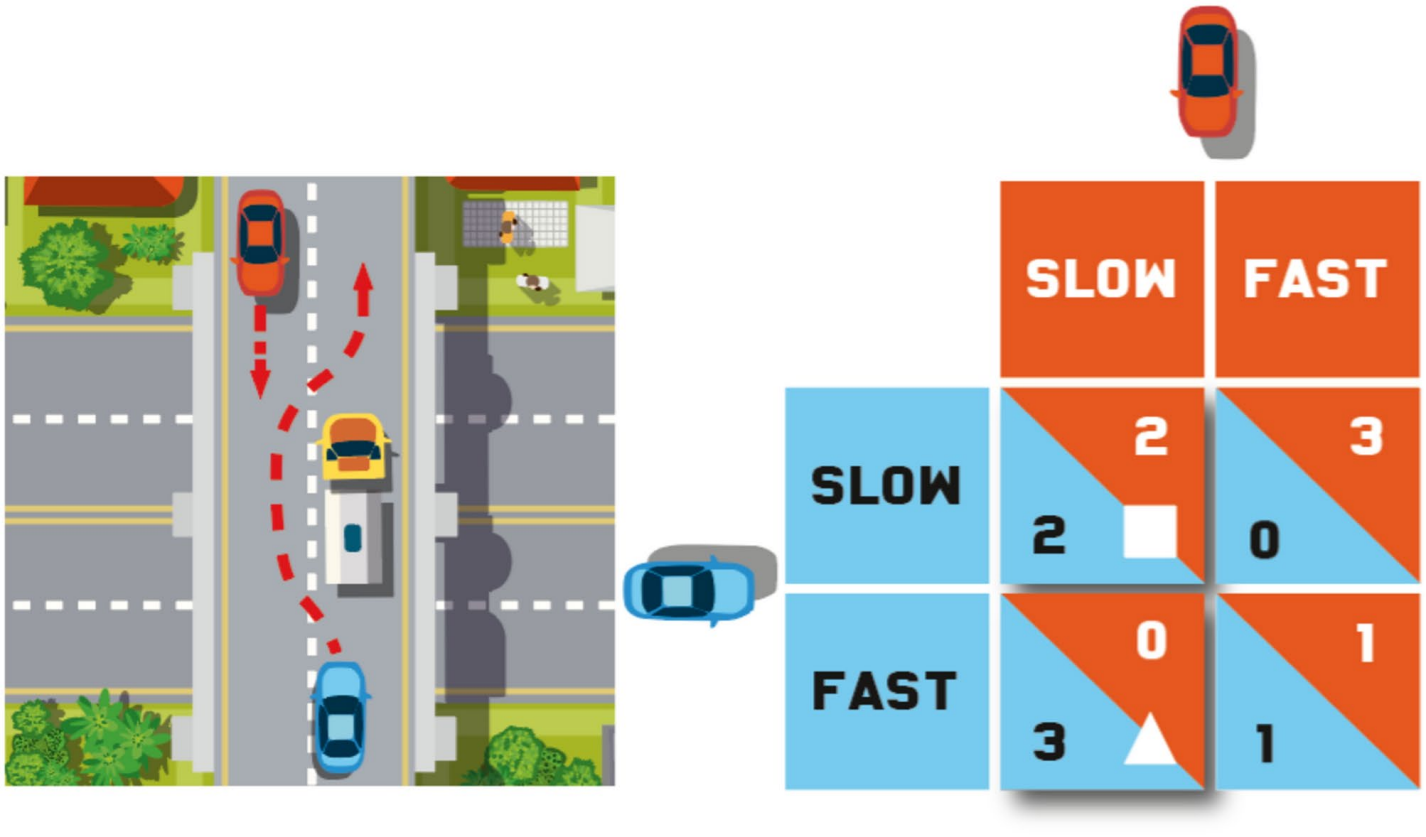
The Prisoner’s Dilemma game on the road. Left: Two vehicles (blue and red) enter a narrow section of a road caused by a broken-down truck. The section is wide enough for both cars to pass one another safely if they both proceed slowly. Both drivers have to quickly decide what to do without being able to explicitly communicate their intentions to one another. What one wants to do depends on how they think the other will react to the situation. If one expects the other to slow down, creating sufficient space on the road to push through, one could push on without reducing speed. This would force the other driver to hit on breaks. If both drivers think this way, the result will be a stalemate. They will both have to hit on breaks and stop. The drivers will pass one another eventually, but the manoeuvers will take longer than it would had they both proceeded slowly to begin with. Right: In the game matrix of this scenario, the driver of the blue car chooses between the two options identified by rows; the driver of the red car—by columns. Their choices jointly determine the outcome and the numbers in each cell are payoffs to the row (blue) and the column (red) player, respectively. The cooperative outcome (identified with the white square) is for both drivers to slow down and pass one another safely. This constitutes a tacit compromise, whereby neither driver attempts to outsmart the other by exploiting a predicted cooperative maneuver (“the other will slow down and swerve”) for one’s personal benefit (“I should push on”). One such exploitative outcome is identified with the white triangle.

Surveys and vignette-based studies, such as the *Moral Machine* study, which, in 2016, asked people from more than 200 countries about their ethical views on the use of fully automated (“self-driving”) cars, warn of important cross-cultural differences in people’s judgements on the implementation and regulation of new technologies stemming from artificial intelligence (AI) research^1^. People’s actual behaviours when they will interact with these new technologies are harder to anticipate. Behavioural game theory offers robust methods to address this question by making it possible to immediately compare human interaction with automated systems to well-documented benchmarks on human interaction with fellow humans across time, interactive contexts, and cultures^2–10^. Game theorists use economic games with monetary incentives to construct scenarios that are easy to implement in behavioural studies, but that also capture the fundamental features of diverse types of interaction from human daily lives. For example, Fig. 1 shows how an everyday traffic situation can be modelled as an instance of the well-known Prisoner’s Dilemma game (for other examples of how game theory can be used to model traffic interactions, see^11–13^).

Comparing human–AI and human–human interactions with the use of behavioural game theory methods can bring valuable insights for future policy, regulation, and design of AI-powered tools and interactive artificial agents. It can also serve an urgent need for more cross-cultural studies of human cognition and behaviour, whether in interactions with fellow humans or with automated, AI-powered systems^14,15^.

Studying how humans interact with AI systems across different cultures can be complex due to varying levels of technology adoption and familiarity in different parts of the world. Japan and the United States are particularly useful for comparison, as both are early adopters and significant developers of AI-powered technologies. For example, Japan leads the world in the supply of industrial robots and is among the top three countries in AI research alongside China and the United States. Toshiba, a large Japanese conglomerate, is a major player in AI-related patents, recently surpassed only by American giants Microsoft and IBM^16^. While Japan and the United States are comparable in terms of AI-related business and research, cultural differences across these countries may impact human behaviour and psychology in people’s interactions with AI, making them interesting candidates for cross-cultural comparison.

Cultural differences may lead to varied reactions towards interactive artificial agents in Japan and the United States. Japan’s historical affinity for animism and the belief that non-living objects can possess souls in Buddhism has led to the assumption that Japanese people are more accepting and caring of robots than individuals in other cultures^17,18^. Recent studies indicate that people in Japan, compared to people in “the West,” have a greater tendency to perceive artificial agents as similar to humans. In Japan, for example, humans and robots are frequently depicted in pictures as partners, whereas in Western societies they are often pictured as distinct or opposing strangers^19^. Additionally, a larger proportion of people in Japan believe that robots can experience and express emotions, as compared to their counterparts in the United States^20^. That said, a recent survey of 50 studies on how culture shapes people’s attitudes towards robots suggests that, while people in Japan and Korea are exposed to robots more, they perceive robots less positively than people in Western cultures, e.g., the United States, Australia, and France (possibly due to having formed more realistic expectations of the robots’ contemporary capabilities)^17^. Yet, such cross-cultural comparisons of people’s explicit and implicit attitudes towards robots do not always generalize^18,21^.

Prior cross-cultural game-theoretic studies found little or no difference in people’s willingness to cooperate with fellow humans in Japan and the United States^22–24^. This applies not only to these two countries: a large recent survey of 1506 studies on human–human cooperation in a series of social dilemma settings (in which people have to choose between how much to cooperate and how much to compete with others in making money) reports little cross-cultural variation in people’s cooperation with fellow humans across 70 distinct societies^9^. That said, compared to Japanese, Americans have been found to be more trusting of strangers in some game-theoretic settings^10^. It is standard in these studies to assess trust behaviourally, for one’s decision to cooperate with others usually requires one to sacrifice some of their personal interests to attain mutually beneficial results and to expect (trust) that others would do so too. In the context of this study, we interpret trust that way as well.

At present, our understanding of human general attitudes towards AI and of their actual cooperation with and trust in fellow humans offers then mixed predictions for how interactions between humans and AI-powered agents—for example, between human traffic participants and the much anticipated fully automated vehicles—may unfold in different parts of the world. Importantly, we focus here not on cases in which people use AI-powered tools to attain their own personal objectives where there are no conflicts of interest between what people want to achieve and what their tools are there to do. Instead, we focus on human interactions with automated agents that are used as tools by someone else, e.g., as it would be the case when a human cyclist encounters someone else’s automated vehicle in busy traffic.

Recently, behavioural scientists have begun to employ game-theoretic methods to analyse human–AI interactions of this kind. These initial studies have consistently shown that when people interact with artificial agents, they tend to act more self-interestedly, prioritizing their personal interests, than when they interact with fellow humans, in which case they often cooperate to attain mutually beneficial results^25^. Part of this reduced cooperation with machines comes with human readiness to exploit artificial agents: even when people expect that machines will be as cooperative with them as fellow humans, they are more willing to take advantage of these machines than they are of cooperative humans^26,27^. The reasons for that can be manifold. For example, people may not treat machines as entities that can be “hurt” from exploitation, or they may not perceive artificial agents’ decision to cooperate with them as an outcome of an internally negotiated compromise when weighting personal versus mutual interests in interactive settings, which has been theorized to explain cooperation among humans^26^. Whatever the reason, the majority of game theory experiments on human–AI interaction in mixed-motive settings (that is, where the interacting parties’ personal and mutual interests are not perfectly aligned) have been carried out with participants recruited in “Western” societies, such as the United States, Canada, and the United Kingdom. In this study, we focus on whether people’s greater willingness to exploit cooperative artificial agents compared to cooperative humans in mixed-motive economic games is also present in Japan. Following recent works^17,21^, we will from now on use the term “culture” to refer simply to people’s country of residence (in our case, Japan and the United States).

Our reviewed prior findings did not suggest precise predictions. None of our surveyed studies carried out a cross-country comparison of people’s *expectations* about their game partner’s cooperation in the studied mixed-motive games. Since prior research revealed little variation in people’s actual *decisions* to cooperate with fellow humans in Japan and the United States and since it is easier to rationalize one’s decision to cooperate when one expects their game partner to cooperate as well, we had reason to expect that people’s *expectations* about their human game partner’s decision to cooperate would be similar across the two cultures as well. With no prior evidence to suggest otherwise, we hypothesized that the same would hold in people’s interactions with artificial agents. Also, since previous experiments revealed little variation across people’s *expectations* about their human and artificial agent partner’s cooperation with them in the United States^26^, we hypothesized that a similar variability would hold in Japan as well. Regarding cross-cultural variation in *algorithm exploitation*—people’s greater willingness to exploit a cooperative artificial agent compared to a cooperative human for selfish gain—prior findings suggested mixed predictions. On the one hand, cross-cultural comparisons of people’s general attitudes toward machines suggested that people in Japan would treat machines similarly to how they treat fellow humans. That should result in a lesser extent of algorithm exploitation in Japan compared to the United States. On the other hand, some previous studies revealed less positive perception of machines among people in Japan compared to people in Western cultures. Taking that into account, we predicted that on balance people in both cultures would show algorithm exploitation, but possibly to a lesser degree in Japan than in the United States.

We pre-registered our hypotheses prior to conducting our study (osf.io/kr8qx). Regarding people’s *expectations*, we hypothesized that participants in Japan would expect artificial agents to be as cooperative as fellow humans (H1) and that Japanese and American participants’ expectations about the cooperativeness of artificial agents would be the same as well (H2). As we realized later, our original wording of H2 (“Participants in Japan expect artificial agents to be as cooperative with them as other humans in the USA.”) was somewhat ambiguous. However, as corroborated by our experiment design (which we also pre-registered), our goal was to elicit and compare participants’ expectations about the cooperativeness of artificial agents and humans within their own country (our goal was not to elicit, for example, a Japanese participant’s expectation about an American participant’s decision to cooperate). Regarding people’s *decisions to cooperate*, we hypothesized that participants in Japan would show algorithm exploitation (H3). We also pre-registered two competing hypotheses: that participants in Japan would show less algorithm exploitation than participants in the United States (H4) and that participants in Japan would show similar levels of algorithm exploitation as participants in the United States (H5).

## Methods

### Overview of games

**Fig. 2:**
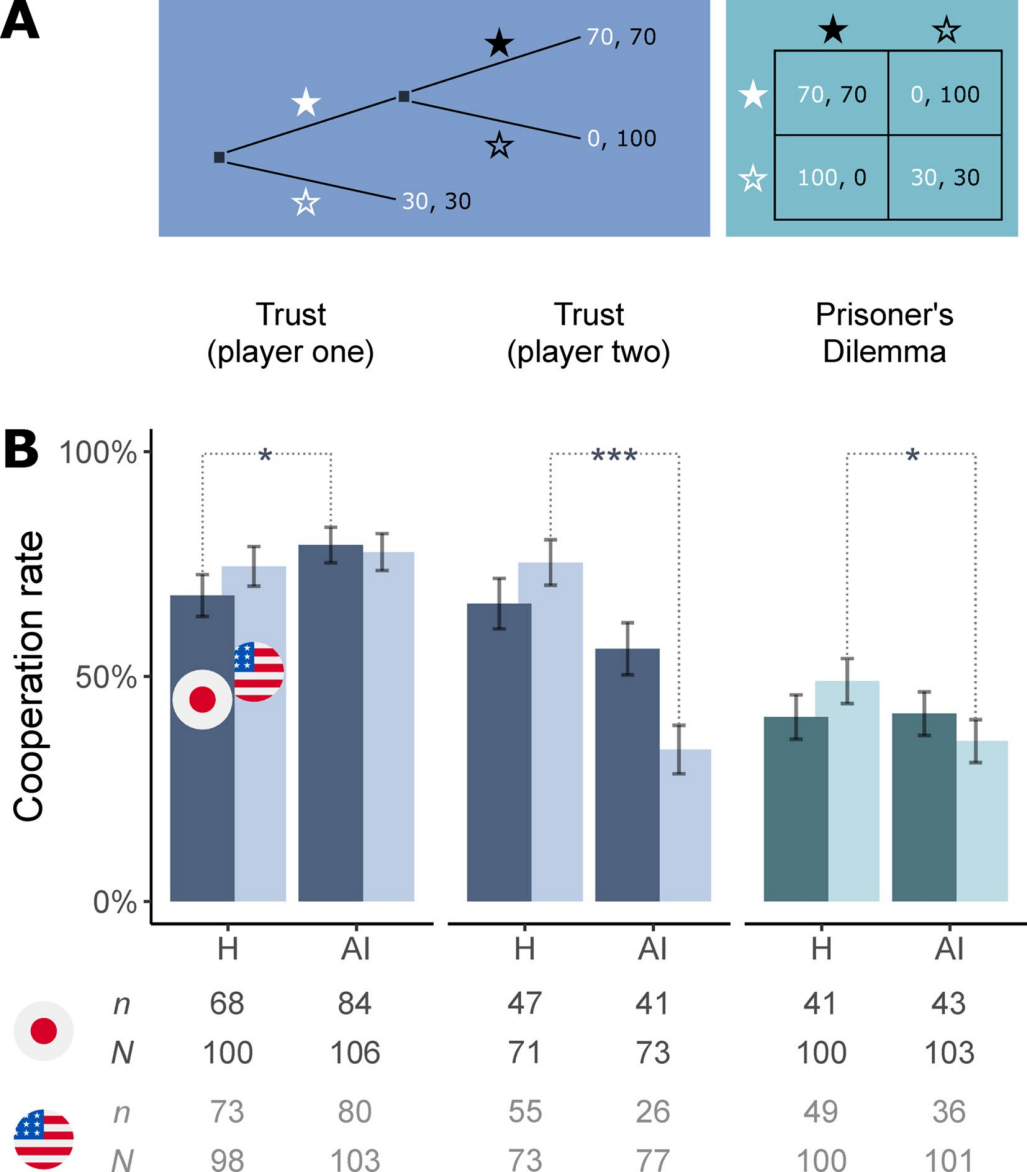
People in Japan cooperate with AI agents as much as they do with humans, whereas Americans cooperate with AI agents less. (**A**) Half of the participants in the Trust game were assigned to the role of player one (choice between the solid and the hollow white star) and the other half to the role of player two (choice between the solid and the hollow black star). The numbers at the three possible outcomes of the game are payoffs to players one and two, respectively (1 point = ¥2). Participants in the Prisoner’s Dilemma game chose between the two options identified by rows. The numbers in each cell are payoffs to the participant and their co-player, respectively. (**B**) Participants’ cooperation rates when they faced a human (H) and AI co-player in Japan (dark columns) and the United States (light columns). Note that not all participants in the role of player two in the Trust game had an opportunity to make a choice (this was conditional on the first player’s decision in the game). Bars: mean ± 1 s.d.. *, ***: *p* < 0.05, *p* < 0.001 in Pearson’s chi square one-sided tests for difference in proportions. Below chart: the number of cooperative choices (*n*) and the total number of observed choices (*N*) in each treatment.

We recruited 600 participants in Japan and compared their choices to a previously reported dataset of 604 participants’ choices in the United States (with demographic characteristics of the two samples being largely similar; Fig. S6). Each participant interacted with either another human or an artificial intelligent (AI) agent in one of two well-known mixed-motive games: the one-shot Trust and Prisoner’s Dilemma economic games. In our previous work, we used these games to reveal the phenomenon of *algorithm exploitation* among participants recruited in the United States^26^. Both games involve two players who, independently and without communicating with one another, choose one of two options, identified as solid and hollow stars (Fig. 2A). Their choices jointly determine the outcome that obtains—a specific distribution of points to the interacting players. These points were converted into monetary earnings for participants, allowing us to incentivize their decisions and make certain game outcomes particularly appealing to individual players.

In the Trust game, the two players make choices one after the other. The first player to make a move can either end the game immediately (play hollow white star) or take a chance on cooperation (play solid white star). Ending the game immediately leaves both players with a small but safe 30 points each. If the first player chooses to cooperate, the second player gets to choose the game’s outcome. They can choose between a cooperative and a selfish outcome (play solid or hollow black star, respectively). The cooperative outcome gives players 70 points each. The selfish outcome gives 100 points to player two and no points to player one. Therefore, it only makes sense for the first player to cooperate if they expect (or trust) the second player to reciprocate. Cooperation for the first player is nevertheless risky because the second player may be tempted to defect (play hollow black star) in order to reap a higher personal payoff (100 instead of 70 points).

In our second game, the Prisoner’s Dilemma, both players make decisions at the same time. The key difference between the Prisoner’s Dilemma and the Trust game is that in the Prisoner’s Dilemma, both players make decisions without knowing what their partner’s choice is. As in the game of Trust, mutual cooperation (both players choosing solid star) is better for both players than mutual defection (both choosing hollow star). Each player, however, has a personal incentive to defect (play hollow star) in order to reap a higher personal payoff when they expect their partner to cooperate (play solid star). In addition to their own choices, we recorded what participants predicted about their human or AI co-players’ choices in these games.

We gave our experiment participants the exact same task instructions and stimuli that were used in the original study, which was conducted with participants recruited in the United States^26^. The only difference was that we translated all English texts from the original study into Japanese and paid our participants in Japanese Yen instead of US Dollars. As in the previous study, we informed the cohort of participants who interacted with an AI agent (i.e., an AI co-player) that this agent was being developed to reason similarly to humans (see Further details concerning methodology below). In the Results section, we will present our findings from Japan alongside the findings from the original study, which included participants recruited in the United States.

### Further details concerning methodology

We set up our interactive experiments online using the LIONESS Lab software^28^ and recruited participants from the online labour market Yahoo! Crowdsourcing Japan. Each subject received ¥50 for their participation and an additional bonus of up to ¥200 based on the payoff they received from playing the assigned game. We chose these amounts to be comparable to those used in the original study^26^, in which participants were recruited in the United States ($0.50 and $2.00, respectively). The exchange rate was ¥1 = $0.009 (Google Finance) when we conducted our experiments in Japan (Jun–Aug 2021). According to *The Economist’s* Big Mac index*,* the Japanese yen was 28% undervalued relative to US Dollar a the time (https://www.economist.com/big-mac-index), making our used exchange rate of ¥1 = $0.01 appropriate for our subsequent comparative analyses.

We used established and recommended procedures^28–30^ to protect the experiments from participation by bots and inattentive subjects, and to prevent subjects from entering our experiments multiple times. As in the original study, our goal was to recruit 100 participants for each role that the participants were assigned to (a player in the Prisoner’s Dilemma and the roles of player one and two in the Trust game) in both the human–human and human–AI treatments. Out of 1,163 subjects who logged into our experiments, 600 completed them successfully (29.7% women, 68.8% men, 0.2% other, and 1.3% of undisclosed gender; mean age ± 1 standard deviation = 45.5 ± 10.5). Dropouts included 217 (19%) who failed our comprehension tests designed to filter out bots and inattentive subjects, which falls within the range (17%–52%) of comparable studies^26,28,29^, and another 346 (30%) who abandoned the experiment before completing the main task or were not matched with another participant in the human–human treatment.

Each subject participated in only one treatment (human–human or human–AI) and played one single-shot Trust or Prisoner’s Dilemma game. Participants began by being given a textual and visual description of the game they would be playing. They were informed that game payoffs would be converted into real money at a rate of 1 point = ¥2. Following the presentation of instructions and examples of how the gameplay would turn out for various combinations of hypothetical choices made by the players, participants took a comprehension test before proceeding to the main task of the experiment. In this test, they were shown two possible outcomes of the game that had been assigned to them based on hypothetical choices that they and their co-players could make. These were distinct from those used in previous examples. Participants were then asked to answer a series of multiple-choice questions about the number of points and monetary earnings that they and their co-players would earn in each of the two outcomes. They had two chances to answer correctly and were not allowed to continue with the experiment if they failed.

The instructions, visual stimuli, and the test procedure used in this study were identical to those used in the original study with participants recruited in the United States^26^. The only difference was that we translated all of the original study’s English texts into Japanese and paid our participants in Japanese Yen instead of US Dollars. To ensure that our translations did not distort the intended meaning of the original instructions presented to participants in English, we used Google Translate to back-translate key sections of instructions into English and adjusted the texts in Japanese as needed. For a diagram of the test procedure and a selection of screenshots, see Fig. S7.

At the start of the experiment, we assigned each subject to a co-player who was either another human participant (in the human–human treatment) or an AI agent (in the human–AI treatment). We informed the participants of the type of their co-player (human or AI). For each game, we first recorded the frequencies of cooperation and defection in the human–human treatment and used those to determine the AI agent’s choices in subsequent interactions with participants in the human–AI treatment. Specifically, the proportion of participants who cooperated with their co-player in the human–human treatment determined the probability with which the AI agent cooperated with its human co-player in the same setting (that is, in the same game and the same player role in the case of the Trust game).

We introduced the AI agent to participants in the human–AI treatment as follows:

*The AI is being developed to be sensitive to outcomes of its decisions similarly to what is found in human population. For example, to be aware of the points that both you and the AI can earn and to realize that the outcome of its decision depends also on your choice.*

This was presented in Japanese as follows:

このAIは，人間と同じように意思決定の結果を気にするように開発されています。例えばAIは，あなたとAIがそれぞれ獲得できる可能性のあるボーナスを意識しています。 さらにAIは，決定の結果が，自身の選択だけでなく，あなたの選択にも依存することも分かっています。

Participants in the human–human treatment were told the following:

*You and another participant who is also online like you will play a game. The other participant is reading the same instructions as you.*

This was presented in Japanese as follows:

他の参加者は, あなたと同じようにオンラインで参加しようとしています。他の参加者もあなたと同じ説明文を読んでいます。

After passing the comprehension test, participants entered a lobby, in which they were matched with a co-player to play the game assigned to them. In the human–human treatment, participants continued to the game as soon as two people were present in the lobby. That meant that half of participants who entered the lobby proceeded to the game almost immediately, while the other half had to wait a little while for another participant to enter the lobby. To create a similar experience, half of participants in the human–AI treatment proceeded to play the game quickly (after a 2 s delay) while the other half had to wait 10 s to be connected to their AI co-player (Fig. S7).

Having entered a game, participants were asked to (1) state their choice (solid or hollow star), (2) state what they expected their co-player’s choice to be (solid or hollow star), and (3) rate their confidence in their prediction on a 6-level Likert scale ranging from 50% (“not at all, this is a random guess”) to 100% (“very confident, certain”). The order of the questions was counterbalanced: half of the participants answered them in the order (1)➤(2)➤(3) (in the choose-predict treatment) and the other half in the order (2)➤(3)➤(1) (in the predict-choose treatment). The exception were the participants in the role of player two in the Trust game who all answered these questions in the order (2)➤(3)➤(1).

After all three questions were answered, the actual outcome of the interaction with the co-player was revealed. Participants were then asked to indicate how happy, relieved, victorious, angry, guilty, and disappointed they felt about the outcome on a 7-level Likert scale ranging from 0 (“not at all”) to 6 (“very”). At the end of the experiment we collected demographic data on subjects’ age, gender, experience with game theory and/or economics disciplines, and religiosity (all provided by participants). We did not collect data on subjects’ race or ethnicity. Fig. S6 shows demographic characteristics of our compared samples from Japan and the United States. We invite you to play the Prisoner’s Dilemma game against an AI co-player (in Japanese) here: https://www.cvbe-experiments.com/JPNPD_demo/_beginParticipant.php.

### Analysis plan

We conducted our analyses in R (version 4.4.0). Based on our pre-registered plan, we used Pearson’s chi-square tests for differences in proportions to compare participants’ rates of cooperation and predicted cooperation across treatments (human–human versus human–AI) and countries. Since *algorithm exploitation*—people’s greater willingness to exploit a cooperative artificial agent compared to a cooperative human for selfish gain—is a one-directional prediction about differences in rates of cooperation, we planned and used one-sided statistical tests to test for it (for example, when testing H3). This replicates the method used in the previously conducted study with participants recruited in the United States^26^. Our discussion of the results will clearly indicate which tests were one-sided. To further corroborate our findings beyond the bar set by pre-registration, especially concerning H4, we conducted binomial logistic regressions with a participant’s decision to cooperate as the dependent variable and a participant’s country, type of co-player, and interaction between the two as independent variables. We also conducted Bayes Factor analyses to investigate H1, H2, and H5. In the exploratory part of our study, which we will explain in more detail when discussing results, we used Wilcoxon–Mann–Whitney tests to compare distributions of participants’ reported emotional states when they exploited a cooperative human or AI co-player. In all cases, the data met the assumptions of the statistical tests used.

### Ethics approval and pre-registration

The University of London School of Advanced Study Research Ethics Committee approved the study after it was reviewed for compliance with ethical research standards (approval ref. SASREC_1819_313A). The procedure was also under the approval of the Waseda University Research Ethics Committee. Prior to collecting data, we pre-registered our experiment design and general data analysis plans on the Open Science Framework database online (osf.io/kr8qx). We obtained informed consent from all participants who took part in our study and we pre-registered our hypotheses on June 11, 2021, prior to conducting our study. The study adhered to the ethical principles of the Declaration of Helsinki.

## Results

### The Trust game

The Trust game was played by 397 participants recruited in Japan, whose choices in the game were compared to choices in a previously obtained dataset of 403 participants recruited in the United States^26^. Each participant was assigned to the role of the first or the second player in the game (Fig. 2A), and their co-player was either a human or an AI agent. The participants who interacted with humans are indicated by H on the horizontal axis in Fig. 2B. In this group, the majority of player one participants cooperated and the majority of player two participants responded in kind (68% and 66%, respectively; dark blue columns in Fig. 2B). These proportions are comparable to those previously observed among participants recruited in the United States (74% and 75%, respectively; light blue columns in Fig. 2B). Although cooperation rates were slightly lower in Japan, the difference across countries was not statistically significant (χ^2^(1) = 1.017, *p* = 0.313, OR = 0.73, 95% CI [0.39, 1.35] and χ^2^(1) = 1.457, *p* = 0.227, OR = 0.64, 95% CI [0.31, 1.32]; two-sided tests).

In Fig. 2B, the participants who interacted with AI agents are indicated by AI on the horizontal axis. In Japan, the majority (79%) of this group of participants cooperated in the role of player one. This proportion was nearly identical in the United States (78%). However, when focusing on participants in the role of player two, we discovered a clear difference in people’s treatment of AI agents across the two countries. Significantly more player two participants cooperated with AI agents in Japan (56%) than did in the United States (34%; χ^2^(1) = 7.606, *p* = 0.006, OR = 2.51, 95% CI [1.3, 4.87]; two-sided test). Although Japanese people cooperated with AI agents somewhat less than they did with humans, this difference was not statistically significant (χ^2^(1) = 1.525, *p* = 0.109, OR = 0.65, 95% CI [0.33, 1.28]; one-sided test). In the United States this difference was significant: less than half as many people cooperated with AI agents as with humans (34% vs. 75%, respectively; χ^2^(1) = 26.077, *p* = 2 × 10^−7^, OR = 0.17, 95% CI [0.08, 0.34]; one-sided test).

**Fig. 3:**
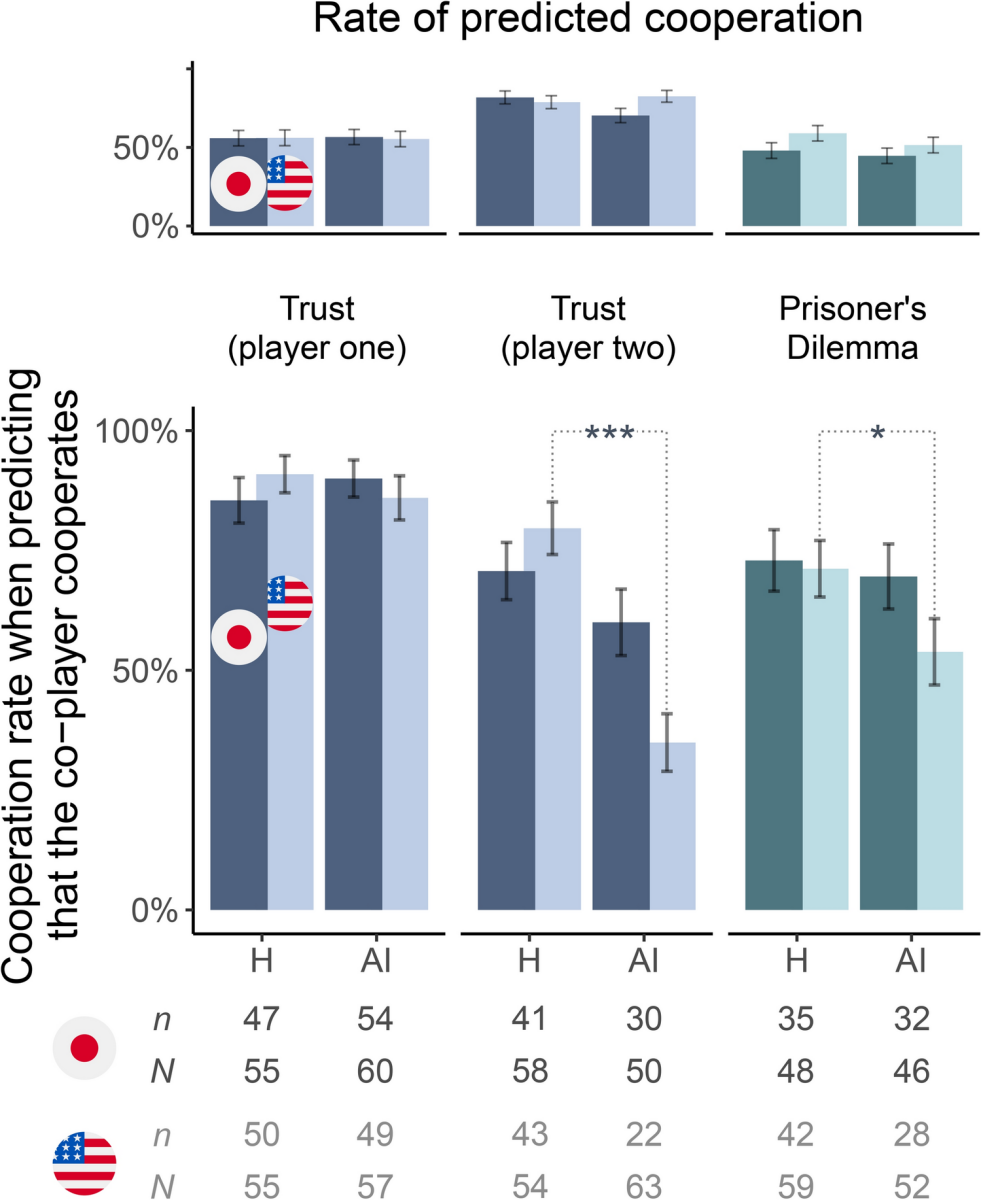
People in Japan and the United States expect AI agents to be as cooperative as humans, but only in Japan they are as likely to reciprocate cooperation with both types of co-player. Top chart: proportions of participants in Japan (dark columns) and the United States (light columns) who predicted that their human (H) or AI co-player would cooperate. Bottom chart: rates of cooperation among participants who predicted that their co-player would cooperate. Not all participants who were assigned to the role of player two and made a prediction about their co-player’s choice in the Trust game had an opportunity to make a choice themselves. (This was conditional on the first player’s decision in the game.) Bars: mean ± 1 s.d.. *, ***: *p* < 0.05, *p* < 0.001 in Pearson’s chi square one-sided tests for difference in proportions. Below chart: the number of cooperative choices (*n*) and the total number of observed choices (*N*) in each treatment.

Figure 3 shows participants’ choices conditioned on their prediction about how their human and AI co-player would behave in the game. Here too, the critical observation to be made is in participants who played the role of player two. In both countries, people predicted that their co-player, be they human or AI, were highly likely to cooperate (top panel in Fig. 3). Participants in Japan expected AI agents to be somewhat less cooperative than humans (70% vs. 82%) and they were less optimistic about AI agents’ cooperativeness compared to participants in the United States (where 83% predicted that AI agents would cooperate). However, among participants who made optimistic predictions, fairly similar proportions cooperated with their AI and human co-player in Japan (60% vs. 71%, respectively; dark blue columns in the bottom panel in Fig. 3; χ^2^(1) = 1.362, *p* = 0.122, OR = 0.62, 95% CI [0.28, 1.38]; one-sided test). In the United States, significantly fewer people cooperated with AI agents than with humans (35% vs. 80%, respectively; light blue columns in the bottom panel in Fig. 3; χ^2^(1) = 23.539, *p* = 6 × 10^−7^, OR = 0.14, 95% CI [0.06, 0.32]; one-sided test).

Overall, these findings suggest that participants in Japan were nearly as likely to cooperate with artificial agents as they were with humans, whereas participants in the United States were significantly more eager to exploit cooperative artificial agents for selfish gain than they were to exploit cooperative humans. In other words, the strong evidence for *algorithm exploitation* that has been observed in the United States^26^, was not observed in Japan. Note that participants’ predictions about their co-player’s cooperation do not in fact matter for drawing this conclusion, since, irrespective of their earlier predictions, people who played the role of player two in the Trust game made choices knowing that their co-player cooperated with them.

**Fig. 4:**
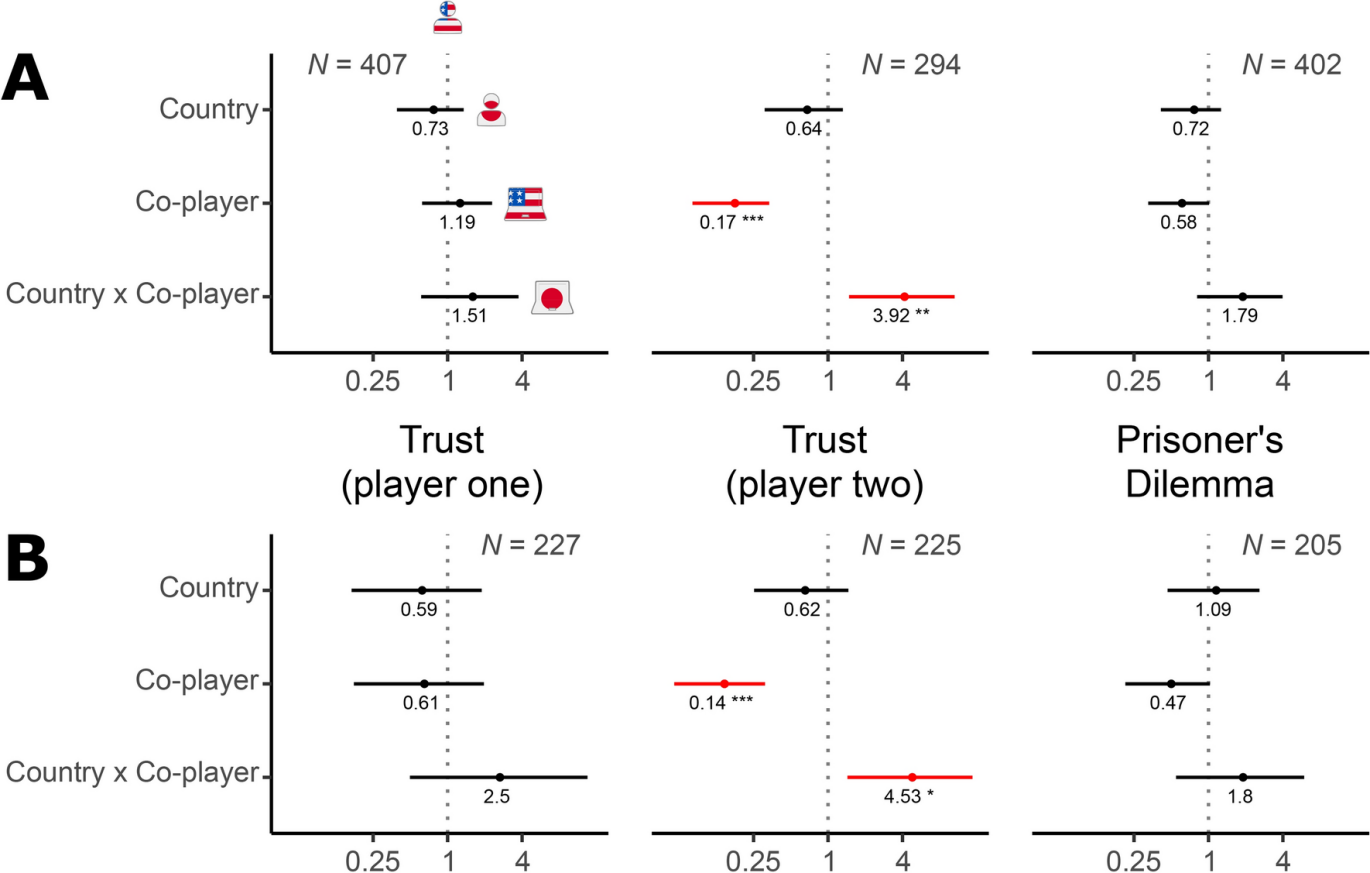
The difference between the odds of cooperating with an AI agent and the odds of cooperating with a human is less pronounced in Japan than it is in the United States. (**A**) The results of a binomial logistic regression with a participant’s decision to cooperate as the dependent variable and a participant’s country, type of co-player, and interaction between the two as independent variables. The (exponentiated) country and co-player coefficient values are odds ratios. They show how the odds of cooperating change in each treatment relative to the odds of cooperating in the baseline treatment: participants in the United States who faced a human co-player. For example, in the Trust game in the United States, 73 participants who played the role of player one cooperated, while 25 defected against a human co-player (see Fig. 1B). As a result, the baseline odds of cooperating are 2.92 (there were 2.92 cooperators for each defector). In Japan, the odds of cooperating among participants who faced a human co-player, therefore, are 2.92 × 0.73 = 2.13 (0.73 is the exponentiated country coefficient value; all values are rounded to 2 decimal places). Similarly, the odds of cooperating among participants in the United States who faced an AI agent are 2.92 × 1.19 = 3.47. The (exponentiated) coefficient value of the interaction term is the ratio of odds ratios. It indicates by how much the odds ratio must be adjusted in order to determine the odds of cooperation among Japanese participants who faced an AI agent as co-player. The odds of cooperating in this treatment are 2.92 × 0.73 × 1.19 × 1.51 = 3.83. A deviation from the dotted line indicates an effect on the decision to cooperate. A shift to the right indicates an increase in cooperation (compared to the baseline) among participants in Japan (country) and/or among participants who faced an AI agent (co-player). Bars: 95% confidence intervals. *N*: number of observations. Statistically significant effects are highlighted in red: *, **, ***: *p* < 0.05, *p* < 0.01, *p* < 0.001. (**B**) The same as above, but limited to the set of participants who predicted that their co-player would cooperate.

To further examine the robustness of our results, we conducted a binomial logistic regression with a participant’s decision to cooperate as the dependent variable and a participant’s country, type of co-player, and interaction between the two as independent variables (Fig. 4). Focusing on the coefficient of the interaction term for participants in the role of player two, the direction and statistical significance of its shift from the baseline (the shift from the dotted vertical line in Fig. 4) suggest that the *difference* between the odds of cooperating with an AI agent and the odds of cooperating with a human *is less pronounced* in Japan than it is in the United States (exponentiated B = 3.921, 95% CI [1.479, 10.554], *p* = 0.006). This effect persisted in an expanded regression model in which we controlled for participants’ gender, age, experience with game theory and/or economics disciplines, and religiosity (Fig. S1; exponentiated B = 4.371, 95% CI [1.614, 12.043], *p* = 0.004, BF = 0.16).

### The Prisoner’s Dilemma game

The Prisoner’s Dilemma game was played by 203 participants recruited in Japan, whose choices in the game were compared to choices in a previously obtained dataset of 201 participants recruited in the United States^26^. As in the Trust game, each participant was paired with either a human or an AI agent as a co-player. In Japan, the proportions of participants who cooperated in both treatments were nearly identical (41% and 42%, respectively; dark turquoise columns in Fig. 2B). This is in sharp contrast with the case in the United States, where significantly fewer people cooperated with AI agents than with humans (36% vs. 49%; light turquoise columns in Fig. 2B; χ^2^(1) = 3.673, *p* = 0.028, OR = 0.58, 95% CI [0.33, 1.01]; one-sided test). Although participants in Japan cooperated with humans somewhat less than participants in the United States, the difference across the countries was not statistically significant (χ^2^(1) = 1.293, *p* = 0.256, OR = 0.72, 95% CI [0.41, 1.27]; two-sided test).

It is important to note that cooperation in the Prisoner’s Dilemma game is risky. If one expects one’s partner to defect (play hollow star), one should defect as well in order to secure a positive payoff in the game (30 instead of 0 points). People in both countries, however, had similar expectations for their human and AI co-players’ decisions. In Japan, 48% expected their human co-player to cooperate and 45% expected an AI agent to cooperate (χ^2^(1) = 0.228, *p* = 0.633, OR = 1.14, 95% CI [0.66, 1.99]; two-sided test; dark turquoise columns in the top panel in Fig. 3). These rates were 59% and 51% in the United States, respectively (light turquoise columns in the top panel in Fig. 3; χ^2^(1) = 1.148, *p* = 0.284, OR = 1.36, 95% CI [0.78, 2.37]; two-sided test). Although the rates of predicted cooperation for both types of co-player were slightly lower in Japan, the difference across countries was not statistically significant (χ^2^(1) = 2.432, *p* = 0.119, OR = 0.64, 95% CI [0.37, 1.12] and χ^2^(1) = 0.952, *p* = 0.329, OR = 0.76, 95% CI [0.44, 1.32]; two-sided tests).

Among participants who predicted that their co-player would cooperate with them, participants in Japan were nearly as likely to cooperate with AI agents as with humans (70% and 73%, respectively; dark turquoise columns in the bottom panel in Fig. 3; χ^2^(1) = 0.129, *p* = 0.360, OR = 0.85, 95% CI [0.35, 2.08]; one-sided test). This was not the case in the United States, where significantly fewer people cooperated with AI agents than with humans (54% vs. 71%; light turquoise columns in the bottom panel in Fig. 3; χ^2^(1) = 3.568, *p* = 0.029, OR = 0.47, 95% CI [0.22, 1.03]; one-sided test). Furthermore, when they predicted that their AI co-player would cooperate with them, people in Japan were less confident about their prediction than people in the United States (Fig. S2). This shows that, compared to participants in the United States, participants in Japan were more cooperative with AI agents despite being less certain about AI agents’ cooperation with them.

In summary, similar to what we discovered from participants’ choices in the Trust game, these findings suggest that participants in Japan were nearly as likely to cooperate with artificial agents as they were with humans, whereas participants in the United States were significantly more eager to exploit cooperative artificial agents for selfish gain than they were to exploit cooperative humans. As in our analysis of participants’ decisions in the Trust game, we conducted a binomial logistic regression with a participant’s decision to cooperate as the dependent variable and a participant’s country, type of co-player, and interaction between the two as independent variables (Fig. 4). Among participants who expected their co-player to cooperate, the shift of the coefficient of the interaction term from the baseline (the shift from the dotted vertical line in Fig. 4) was not statistically significant (exponentiated B = 1.798, 95% CI [0.547, 5.940], *p* = 0.334). However, the direction of the shift was the same as that observed earlier in our analysis of player two participants’ choices in the Trust game. This effect remained the same in an expanded regression model that included additional demographic variables (Fig. S1; exponentiated B = 2.070, 95% CI [0.612, 7.027], *p* = 0.240; BF = 1.24).

### Both games combined

Pooling the results from both games shows that participants in Japan expected AI agents to be slightly less cooperative than fellow humans (57% vs. 61%), but the difference was not statistically significant (χ^2^(1) = 0.960, p = 0.327, OR = 0.85, 95% CI [0.61, 1.18]; two-sided test). They were also somewhat less optimistic about AI agents’ cooperativeness compared to participants in the United States (57% vs. 63%). This difference also was not statistically significant (χ^2^(1) = 2.391, p = 0.122, OR = 0.78, 95% CI [0.56, 1.07]; two-sided test). Respectively, Bayes Factor analyses suggested moderate evidence in support of H1 (BF = 4.16) and anecdotal evidence in support of H2 (BF = 2).

Among participants in Japan who played the role of player two in the Trust game and those who predicted that their co-player would cooperate with them in the Prisoner’s Dilemma, fewer people cooperated with AI than with human co-players (61% vs. 69%), but this difference was not statistically significant (χ^2^(1) = 1.499, p = 0.111, OR = 0.72, 95% CI [0.42, 1.22]; one-sided test). Therefore, testing for H3, we cannot reject the null hypothesis that there is *no* algorithm exploitation in Japan.

As previously, we conducted a binomial logistic regression with a participant’s decision to cooperate as the dependent variable and a participant’s country, type of co-player, and interaction between the two as independent variables. The direction and statistical significance of the shift of the interaction term from the baseline suggest that the *difference* between the odds of cooperating with an AI agent and the odds of cooperating with a human *is less pronounced* in Japan than it is in the United States (exponentiated B = 2.756, 95% CI [1.308, 5.834], *p* = 0.008), lending support to the conclusion that people in Japan show less algorithm exploitation than people in the United States (H4). Correspondingly, Bayes Factor analysis suggested moderate evidence against H5 (BF = 0.21).

### Emotions in the Trust and the Prisoner’s Dilemma games

In exploratory part of our study, to gain insight as to why the extent of *algorithm exploitation* may potentially differ across countries, we administered a short questionnaire after the outcome of a game was revealed to participants (as was done also in the previously conducted study with participants recruited in the United States^26^). We elicited the participants’ emotional reactions—the extents of guilt, anger, disappointment, happiness, victoriousness, and relief—to their achieved outcome of a game using 7-level Likert scales, ranging from 0 (“not at all”) to 6 (“very”). While we did not have specific prior hypotheses regarding these data, we focus the analysis here on defectors in the two games whose co-players cooperated with them (note that in the Trust game these could only be participants who played the role of player two). In other words, we look at how participants in the two countries felt about having exploited a cooperative human or AI co-player (in either of the two games) for selfish gain.

**Fig. 5:**
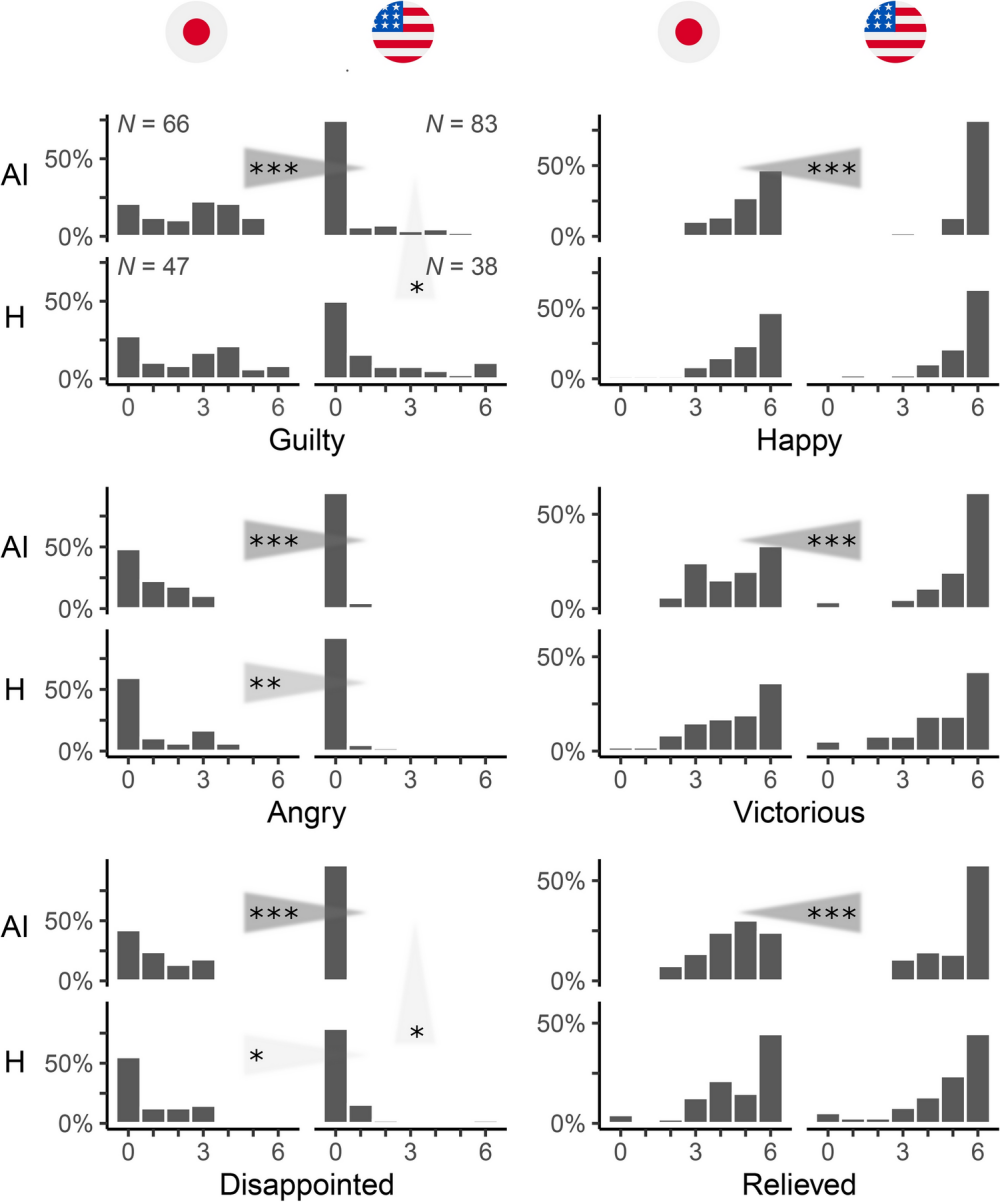
People in Japan feel worse than people in the United States about exploiting an AI agent. The relative frequencies of participants’ reported levels of emotion—guilt, anger, disappointment, happiness, victoriousness, and relief—concerning the outcome of a game that they achieved, as measured by a 7-level Likert scale ranging from 0 (“not at all”) to 6 (“very”). The results shown are for participants who exploited their co-player in a game, which includes those participants who defected in the Prisoner’s Dilemma game when their co-player cooperated and those who defected in the Trust game when they played the role of player two. The distributions on the left for each emotion are of reported levels of emotion among participants recruited in Japan; the distributions on the right are of reported levels of emotion among participants recruited in the United States. The distributions for interactions with AI and human (H) co-players are top and bottom distributions, respectively. The triangular fans indicate a statistically significant proclivity to report a greater level of emotion: *, **, ***: *p* < 0.05, *p* < 0.01, *p* < 0.001 in two-sided Wilcoxon-Mann–Whitney tests for difference in reported levels of emotion, adjusted using the sequentially rejective Bonferroni procedure recommended by Holm^31^ to account for multiple testing (namely, one test for each emotion). The number of responses (*N*) in each treatment, displayed in the top-left panel, is the same for all elicited emotions.

Defectors who exploited their AI co-player in Japan reported feeling significantly more guilty (M_JP_ = 2.5, SD_JP_ = 1.7, M_US_ = 0.7, SD_US_ = 1.4, W = 4297.5, *p* = 7 × 10^−10^, rank-biserial *r* = 0.57, 95% CI [0.43, 0.68]; two-sided test), more angry (M_JP_ = 0.9, SD_JP_ = 1.0, M_US_ = 0.1, SD_US_ = 0.3, W = 4019, *p* = 9 × 10^−10^, rank-biserial *r* = 0.47, 95% CI [0.31, 0.60]), more disappointed (M_JP_ = 1.1, SD_JP_ = 1.2, M_US_ = 0.1, SD_US_ = 0.6, W = 4220, *p* = 3 × 10^−12^, rank-biserial *r* = 0.54, 95% CI [0.40, 0.66]), less happy (M_JP_ = 5.1, SD_JP_ = 1.1, M_US_ = 5.7, SD_US_ = 0.9, W = 1736.5, *p* = 1 × 10^−5^, rank-biserial *r* = − 0.37, 95% CI [− 0.52, − 0.19]), less victorious (M_JP_ = 4.5, SD_JP_ = 1.4, M_US_ = 5.2, SD_US_ = 1.3, W = 1787, *p* = 0.0002, rank-biserial *r* = − 0.35, 95% CI [− 0.50, − 0.17]), and less relieved (M_JP_ = 4.5, SD_JP_ = 1.2, M_US_ = 5.1, SD_US_ = 1.3, W = 1902, *p* = 0.0008, rank-biserial *r* = − 0.31, 95% CI [− 0.46, − 0.13]) than did defectors in the United States (Fig. 5; compare the plots horizontally in rows labelled “AI” for each emotion). Briefly put, Japanese participants felt significantly worse about exploiting an artificial agent than did Americans. (Since we performed six tests to report this overall finding—namely, one test for each emotion in Fig. 5—we adjusted the *p* values using the sequentially rejective Bonferroni procedure recommended by Holm^31^.) This result does not appear to be simply due to Japanese participants’ unwillingness to report extreme levels of emotion on the Likert scales used to elicit their emotional states. Reported levels of emotion in cases where they and their co-players cooperated suggests that Japanese participants were just as eager as American participants to report extreme levels of emotion in different circumstances (Fig. S3).

Our second observation is that people in Japan felt similarly about exploiting both types of co-player across all surveyed emotions, whereas people in the United States felt significantly more guilty (M_H_ = 1.5, SD_H_ = 2.1, M_AI_ = 0.7, SD_AI_ = 1.4, W = 1177.5, *p* = 0.038, rank-biserial *r* = 0.25, 95% CI [0.04, 0.45]; two-sided test) and more disappointed (M_H_ = 0.4, SD_H_ = 1.1, M_AI_ = 0.1, SD_AI_ = 0.6, W = 1303, *p* = 0.013, rank-biserial *r* = 0.17, 95% CI [− 0.05, 0.38]) when they exploited a human than when they exploited an AI agent (Fig. 5; compare the plots vertically in columns associated with responses in Japan and the United States for each emotion; we adjusted the *p* values to account for multiple testing as described above).

**Fig. 6:**
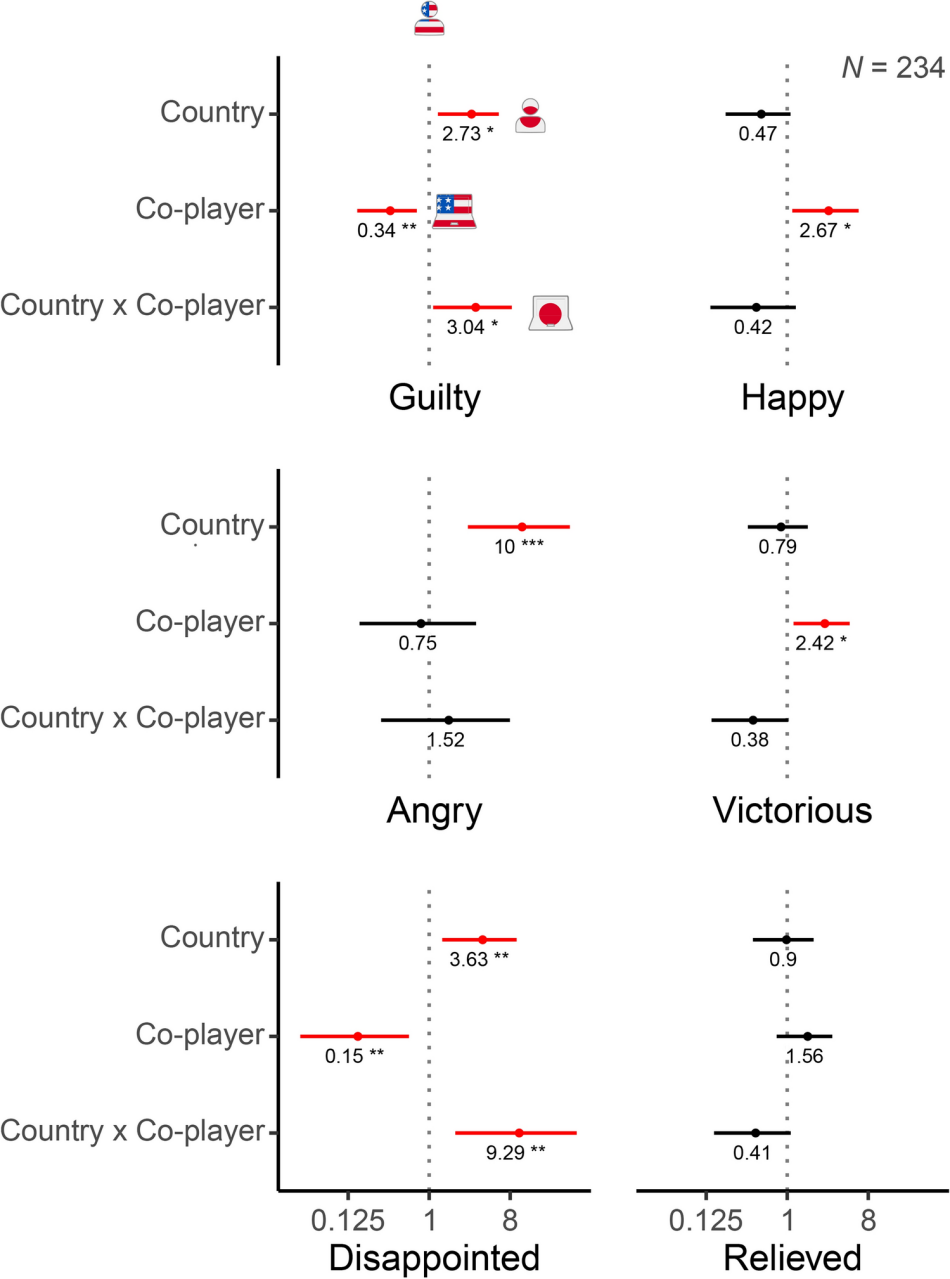
The difference between the odds of feeling worse about exploiting a co-player in Japan and the odds of feeling worse about exploiting a co-player in the United States is more pronounced in people’s interactions with AI agents than in their interactions with fellow humans. The results of ordinal logistic regressions with a participant’s proclivity to report a higher level of emotion (guilt, anger, disappointment, happiness, victoriousness, and relief) as the dependent variable and a participant’s country, type of co-player, and interaction between the two as independent variables. The results shown are for participants who exploited their co-player in a game, which includes those participants who defected in the Prisoner’s Dilemma game when their co-player cooperated and those who defected in the Trust game when they played the role of player two. The (exponentiated) country and co-player coefficient values are odds ratios. They show how the odds of reporting a higher level of emotion change in each treatment relative to the odds of reporting a higher level of emotion in the baseline treatment: participants in the United States who faced a human co-player. The (exponentiated) coefficient value of the interaction term is the ratio of odds ratios. It indicates by how much the odds ratio must be adjusted in order to determine the odds of reporting a higher level of emotion among participants in Japan who faced an AI agent as co-player. A deviation from the dotted line indicates an effect on the reported level of emotion. A shift to the right indicates a tendency to report a higher level of emotion (compared to the baseline) among participants in Japan (country) and/or among participants who faced an AI agent (co-player). Bars: 95% confidence intervals. Statistically significant effects are highlighted in red: *, **, ***: *p* < 0.05, *p* < 0.01, *p* < 0.001. The number of observations (*N*) displayed in the top-right panel is the same for all elicited emotions.

Finally, compared to Americans, Japanese people felt significantly more angry (M_JP_ = 1.0, SD_JP_ = 1.4, M_US_ = 0.1, SD_US_ = 0.4, W = 1200.5, *p* = 0.002, rank-biserial *r* = 0.34, 95% CI [0.11, 0.54]; two-sided test) and more disappointed (M_JP_ = 1.0, SD_JP_ = 1.4, M_US_ = 0.4, SD_US_ = 1.1, W = 1140, *p* = 0.048, rank-biserial *r* = 0.28, 95% CI [0.04, 0.49]) about exploiting also a human co-player. However, the differences in participants’ tendency to feel worse about exploiting a co-player across the two countries were more pronounced in their interactions with AI agents (Fig. 5; compare the plots horizontally in rows labelled “AI” and “H” for each emotion). To back up this finding, we conducted ordinal logistic regressions with a participant’s tendency to report a higher level of emotion (guilt, anger, disappointment, happiness, victoriousness, and relief) as the dependent variable and a participant’s country, type of co-player, and interaction between the two as independent variables (Fig. 6). The key is the extent and the direction of the shift from the baseline (for each emotion in Fig. 6, the shift from the dotted vertical line) of the coefficient of the interaction term in these regressions. Put simply, the interaction term measures the difference across the two countries in differences about feeling bad about exploiting one’s human vs. AI co-player. For 2 of the 6 emotions, the shift of the coefficient of the interaction term is statistically significant at 5% significance level (exponentiated B for guilt = 3.039, 95% CI [1.107, 8.341], *p* = 0.031; exponentiated B for disappointment = 9.294, 95% CI [1.955, 44.183], *p* = 0.005). For 2 additional emotions, it is statistically significant at 10% significance level (exponentiated B for victoriousness = 0.384, 95% CI [0.143, 1.026], *p* = 0.056; exponentiated B for relief = 0.409, 95% CI [0.153, 1.096], *p* = 0.075). In all cases, its direction suggests that the difference between the odds of feeling bad about having exploited one’s co-player in Japan and the odds of feeling bad about having exploited one’s co-player in the United States is more pronounced in people’s interactions with AI agents than in their interactions with humans. These effects remained largely the same in expanded regression models that included additional demographic variables to account for participants’ gender, age, experience with game theory and/or economics disciplines, and religiosity (Fig. S4; exponentiated B for guilt = 2.572, 95% CI [0.920, 7.186], *p* = 0.072; exponentiated B for disappointment = 11.147, 95% CI [2.270, 54.735], *p* = 0.003; exponentiated B for victoriousness = 0.389, 95% CI [0.143, 1.062], *p* = 0.065; exponentiated B for relief = 0.441, 95% CI [0.162, 1.201], *p* = 0.109).

## Discussion

There is ample enthusiasm among companies, media, and techno-optimists regarding the potential of AI to enhance human societies. In those idealized visions, people exhibit levels of cooperation with artificial agents that are comparable to, or even surpass, those observed in human–human interactions. Some studies support these visions and show, for instance, that the inclusion of a bot when groups of two or more humans face collective decision problems can actually enhance cooperation and coordination between humans^32,33^. Moreover, in repeated interactions, machines powered by AI can induce cooperative behaviour in humans who are uncertain whether their interaction partner is another human or an artificial agent^34^. However, as things stand, people cooperate with AI agents significantly less than with humans if they know who (or what) they interact with^35^. This is consistent with the previously reported phenomenon of *algorithm exploitation*^26,27^.

Our present findings temper the generalization of these results and show that *algorithm exploitation* is not a cross-cultural phenomenon. Previous surveys and vignette-based studies highlight that people’s general and somewhat hypothetical attitudes towards robots can differ across cultures^17–20,36,37^. Our findings show that cross-cultural differences exist also in how people already treat artificial agents. In line with previous game theory experiments, we found no significant difference in the willingness of Americans and Japanese people to cooperate with other humans. We also discovered no significant cross-cultural differences in people’s expectations of their human or AI co-players’ willingness to cooperate with them (across the two studied games, our analyses suggested anecdotal to moderate evidence in support of H1 and H2). However, the tendency to exploit cooperative artificial agents, which is a marked feature in the United States^26^ and the United Kingdom^27^, was significantly weaker in Japan. Testing for H3, we could not reject the null hypothesis that there is *no* algorithm exploitation in Japan. Compared to participants in the United States, participants in Japan showed less algorithm exploitation in the Trust game (H4), but we could not reject the null hypothesis that people showed similar levels of algorithm exploitation in the Prisoner’s Dilemma. Correspondingly, we found moderate evidence against H5 in the Trust game and only anecdotal evidence in support of H5 in the Prisoner’s Dilemma. We discovered no other systematic differences in people’s willingness to cooperate with human or AI co-players in Japan and the United States based on people’s gender, age, experience with game theory and/or economics disciplines, and religiosity (Fig. S5). That suggests that cultural factors related to people’s country of residence are more important in people’s differential treatment of artificial agents than the other demographic factors which we considered here.

Moreover, we discovered a possible explanation for why people in the United States exploit algorithms more. Although our study design and analysis allow us to draw only a correlational conclusion, Japanese people’s stronger negative emotional response to exploiting cooperative AI agents may temper their willingness to exploit them. A recent study that used a similar version of the Trust game to ours discovered that, when people in the United States interacted with human co-players, they felt more intense emotions compared to when they interacted with computer co-players^38^. This is consistent with our observations that people in the United States have different attitudes toward exploiting cooperative humans and cooperative artificial agents. Similarly, people in China have been reported to perceive mistreatment of automated vehicles in traffic to be more acceptable than mistreatment of human-driven vehicles^39^.

Unlike the findings from the United States and, tentatively, China, we discovered that Japanese people felt similarly about exploiting human and AI co-players. According to a recent study, people in Japan are more likely than people in the United States to believe that robots can experience emotions such as fear, pleasure, and pain. Furthermore, the same study discovered that, when compared to Americans, Japanese people are more willing to accept robots as targets of human moral judgement^20^. This suggests that Japanese people may be more likely than Americans to consider robots to be moral patients—entities to which humans owe certain moral obligations, though this hypothesis still needs to be investigated.

Overall, our findings highlight that the debated benefits and risks of AI for human society already need strong cultural qualifications. Besides mapping the risks posed to vulnerable groups, notably in terms of bias, there is a need to understand and measure cultural or group differences in people’s treatment of artificial agents. If Americans are eager to exploit delivery drones or cooperative fully automated cars on the road, for example, the adoption of such technologies may be slower and riskier than originally anticipated. The implementation of such interactive AI systems may backfire because individuals, who were supposed to cooperate with them, will eagerly exploit them. There may also be spillover effects from interactions with artificial agents to interactions with fellow humans. While much may be debated in favour of caution, one needs to consider critically also how much of these risks belong to a specific culture and/or country. According to our findings, there may be less concern for the introduction of interactive AI agents in Japan because, in mixed-motive settings, in which the interacting parties’ interests are not always perfectly aligned, as it is indeed the case in many day-to-day traffic interactions (Fig. 1), people in Japan are likely to treat artificial agents similarly to how they treat fellow humans—crucially, they are not likely to exploit them. Whether findings from behavioural studies with the economic games of Trust and Prisoner’s Dilemma prove to be ecologically valid in the field is of course another matter. If time shows that they are, there are compelling reasons to believe that fully automated taxis, the success of which on roads will rely on human traffic participants’ cooperation with them, will gain traction in Tokyo before they do in New York.

## Limitations

Our primary objective was to investigate whether *algorithm exploitation* is a cross-cultural phenomenon. When testing for H3, we cannot reject the null hypothesis that there is *no* algorithm exploitation in Japan. Our post-hoc power analysis revealed that our study had 23% power to detect the effect size (Cohen’s *ω*) derived from our chi-square statistic in the Trust and Prisoner’s Dilemma games combined. This means that our study had a relatively low chance of detecting an effect of the observed size under the assumption that such an effect truly exists in Japan. However, and more importantly, we found that people in Japan showed *less* algorithm exploitation that people in the United States (H4) with moderate evidence *against* the hypothesis that people in both countries showed similar levels of algorithm exploitation (H5).

The points earned by the artificial agent in the Trust and the Prisoner’s Dilemma games in our experiments did not materially benefit any third party. Importantly, they did not benefit any human and we did not suggest to our participants that they might. In this light, it is noteworthy that people in Japan were just as eager to cooperate with AI agents as they were with fellow humans, despite the possibility to earn more money by defecting. Even so, it is unlikely that we will ever create and interact with artificial agents that exist solely for their own sake, that is, without ultimately benefiting some human further down the line. According to recent research, people are more likely to share their payoff with a computer co-player when they know that the computer’s payoff directly determines the monetary payoff of another human^40,41^. Relatedly, people’s reliance on artificial agents, for example, their likelihood to use recommendations issued by those agents to inform their own decisions, depends on how those agents are presented and described^42^. We hypothesize that social distance between a human who interacts with an artificial agent and another human who ultimately benefits (or does not benefit) from that artificial agent’s success (or failure) will play a significant role in people’s willingness to cooperate with that agent and their feelings about exploiting it. Future research on this topic will be beneficial.

Our discovery that Japanese people feel worse than Americans about exploiting cooperative artificial agents offers a possible explanation for why *algorithm exploitation* manifests in the United States but not in Japan. One study we reviewed suggests that this may be so because Japanese people are more likely than Americans to believe that robots can experience emotions and are more willing to accept robots as targets of human moral judgement^20^. These hypothesized explanations, however, need to be empirically investigated further and should be treated as speculation for the time being. In particular, our reported association between people’s emotional responses to exploiting cooperative artificial agents and their willingness to exploit those agents is thus far correlational, and future research could investigate whether the link is indeed causal. Further research will be also fruitful to test alternative hypotheses for why people in Japan feel bad about exploiting cooperative artificial agents. One possibility is that, compared to people in the United States, people in Japan are more concerned about their reputation, even when they interact with non-human, artificial agents. Another reason for why people in Japan feel worse about exploiting cooperative artificial agents may be that people in Japan are more likely to imagine and think about some human who stands to benefit (or lose) from the artificial agent’s performance in a game.

While in this paper we used the term “culture” to refer simply to people’s country of residence, *culture* is a much more complex concept. The behavioural differences in people’s treatment of artificial agents that we found across Japan and the United States may be due to many factors, for example, differences in language, economic and political circumstances, and other. Not all such differences may be attributed to differences in cultures. Future research will undoubtedly uncover more fine-grained explanations for our reported people’s behavioural differences across countries. We also invite researchers from other countries to participate in this project to expand our understanding of the underpinnings of cooperation in human–human and human–AI interactions across the globe.

## Supplementary Information

Supplementary Information.

## Data Availability

All data (from Japan and the United States) and statistical analyses that support the findings of this study are publicly available in Open Science Framework at https://osf.io/w6m9h/.

## Code availability

The R code used for the statistical analyses is available in Open Science Framework at https://osf.io/w6m9h/.

## References

[1] Awad, E. et al. The moral machine experiment. *Nature* **563**, 59–64. 10.1038/s41586-018-0637-6 (2018). 30356211 10.1038/s41586-018-0637-6

[2] Colman, A. M. *Game Theory & Its Applications in the Social and Biological Sciences* (Routledge, 1999).

[3] Battalio, R., Samuelson, L. & Van Huyck, J. Optimization incentives and coordination failure in laboratory Stag Hunt games. *Econometrica* **69**, 749–764 (2001).

[4] Camerer, C. F. *Behavioral Game Theory: Experiments in Strategic Interaction* (Princeton University Press, 2003).

[5] McCabe, K. A., Rigdon, M. L. & Smith, V. L. Positive reciprocity and intentions in Trust games. *J. Econ. Behav. Organ.* **52**, 267–275. 10.1016/S0167-2681(03)00003-9 (2003).

[6] Johnson, N. D. & Mislin, A. A. Trust games: A meta-analysis. *J. Econ. Psychol.* **32**, 865–889. 10.1016/j.joep.2011.05.007 (2011).

[7] Rand, D. G., Greene, J. D. & Novak, M. A. Spontaneous giving and calculated greed. *Nature* **489**, 427–430. 10.1038/nature11467 (2012). 22996558 10.1038/nature11467

[8] Rubinstein, A. & Salant, Y. “Isn’t everyone like me?”: On the presence of self-similarity in strategic interactions. *Judgment Decis. Making* **11**, 168–173. 10.1017/S1930297500007270 (2016).

[9] Spadaro, G. et al. Cross-cultural variation in cooperation: A meta-analysis. *J. Personality Social Psychol.* **123**, 1024–1088. 10.1037/pspi0000389 (2022). 10.1037/pspi0000389 35286118

[10] Macy, M. M. and Sato, Y. (2002). Trust, cooperation, and market formation in the U.S. and Japan. *PNAS* 99, No. suppl_3, 7214–7220. 10.1073/pnas.082097399 10.1073/pnas.082097399 PMC128588 12011400

[11] Chater, N., Misyak, J., Watson, D., Griffiths, N. & Mouzakitis, A. Negotiating the traffic: Can cognitive science help make autonomous vehicles a reality?. *Trends Cognit. Sci.* **22**, 93–95. 10.1016/j.tics.2017.11.008 (2018). 29249603 10.1016/j.tics.2017.11.008

[12] Millard-Ball, A. Pedestrians, autonomous vehicles, and cities. *J. Planning Educ. Res.* **38**, 6–12. 10.1177/0739456X16675674 (2018).

[13] Bitar, I., Watling, D., and Romano, R. (2022). How can autonomous road vehicles coexist with human-driven vehicles? An evolutionary-game-theoretic perspective. in *Proceedings of the 8th International Conference on Vehicle Technology and Intelligent Transport Systems—VEHITS,* 376–383. 10.5220/0011079500003191

[14] Barrett, H. C. Towards a cognitive science of the human: Cross-cultural approaches and their urgency. *Trends Cognit. Sci.* **24**, 620–638. 10.1016/j.tics.2020.05.007 (2020). 32534836 10.1016/j.tics.2020.05.007

[15] Henrich, J., Heine, S. J. & Norenzayan, A. Most people are not WEIRD. *Nature* **466**, 29. 10.1038/466029a (2010). 20595995 10.1038/466029a

[16] Dirksen, N. and Takahashi, S. (2020). Artificial intelligence in Japan 2020. *The Netherlands Enterprise Agency*. https://www.rvo.nl/sites/default/files/2020/12/Artificial-Intelligence-in-Japan-final-IAN.pdf.

[17] Lim, V., Rooksby, M. & Cross, E. S. Social robots on a global stage: Establishing a role for culture during human-robot interaction. *Int. J. Social Robot.* **13**, 1307–1333. 10.1007/s12369-020-00710-4 (2020).

[18] Yam, K. C., Tan, T., Jackson, J. C., Shariff, A. & Gray, K. Cultural differences in people’s reactions and applications of robots, algorithms, and artificial intelligence. *Manag. Organ. Rev.* **19**, 859–875. 10.1017/mor.2023.21 (2023).

[19] Sakura, O. Robot and *ukiyo-e* implications to cultural varieties in human-robot relationships. *AI Society* **37**, 1563–1573. 10.1007/s00146-021-01243-8 (2021).

[20] Komatsu, T., Malle, B. F., and Scheutz, M. (2021). Blaming the reluctant robot: Parallel blame judgments for robots in moral dilemmas across U.S. and Japan. In *HRI ’21: Proceedings of the 2021 ACM/IEEE International Conference on Human-Robot Interaction*, 63–72. 10.1145/3434073.3444672

[21] Diana, F. et al. A cross-cultural comparison on implicit and explicit attitudes towards artificial agents. *Int. J. Social Robot.* **15**, 1439–1455. 10.1007/s12369-022-00917-7 (2023). 10.1007/s12369-022-00917-7 PMC10465401 37654700

[22] Brandts, J., Saijo, T. & Schram, A. How universal is behavior? A four country comparison of spite and cooperation in voluntary contribution mechanisms. *Public Choice* **119**, 381–424 (2004).

[23] Ishii, K. & Kurzban, R. Public goods games in Japan. *Hum. Nat.* **19**, 138–156. 10.1007/s12110-008-9034-4 (2008). 26181461 10.1007/s12110-008-9034-4

[24] Kocher, M. G., Cherry, T., Kroll, S., Netzer, R. J. & Sutter, M. Conditional cooperation on three continents. *Econ. Lett.* **101**, 175–178. 10.1016/j.econlet.2008.07.015 (2008).

[25] March, C. Strategic interactions between humans and artificial intelligence: Lessons from experiments with computer players. *J. Econ. Psychol.* **87**, 102426. 10.1016/j.joep.2021.102426 (2021).

[26] Karpus, J., Krüger, A., Verba, J. T., Bahrami, B. & Deroy, O. Algorithm exploitation: Humans are keen to exploit benevolent AI. *iScience* **24**, 102679. 10.1016/j.isci.2021.102679 (2021). 34189440 10.1016/j.isci.2021.102679 PMC8219775

[27] Upadhyaya, N. & Galizzi, M. M. In bot we trust? Personality traits and reciprocity in human-bot trust games. *Front. Behav. Econ.* **2**, 1164259. 10.3389/frbhe.2023.1164259 (2023).

[28] Giamattei, M., Yahosseini, K. S., Gächter, S. & Molleman, L. LIONESS Lab: A free web-based platform for conducting interactive experiments online. *J. Econ. Sci. Assoc.* **6**, 95–111. 10.1007/s40881-020-00087-0 (2020).

[29] Arechar, A. A., Gächter, S. & Molleman, L. Conducting interactive experiments online. *Exp. Econ.* **21**, 99–131. 10.1007/s10683-017-9527-2 (2018). 29449783 10.1007/s10683-017-9527-2 PMC5807491

[30] Horton, J. J., Rand, D. G. & Zeckhauser, R. J. The online laboratory: Conducting experiments in a real labor market. *Exp. Econ.* **14**, 399–425. 10.1007/s10683-011-9273-9 (2011).

[31] Holm, S. A simple sequentially rejective multiple test procedure. *Scand. J. Stat.* **6**, 65–70 (1979).

[32] Shirado, H. & Christakis, N. A. Locally noisy autonomous agents improve global human coordination in network experiments. *Nature* **545**, 370–374. 10.1038/nature22332 (2017). 28516927 10.1038/nature22332 PMC5912653

[33] Shirado, H. & Christakis, N. A. Network engineering using autonomous agents increases cooperation in human groups. *iScience* **23**, 101438. 10.1016/j.isci.2020.101438 (2020). 32823053 10.1016/j.isci.2020.101438 PMC7452167

[34] Crandall, J. W. et al. Cooperating with machines. *Nat. Commun.* **9**, 233. 10.1038/s41467-017-02597-8 (2018). 29339817 10.1038/s41467-017-02597-8 PMC5770455

[35] Ishowo-Oloko, F. et al. Behavioural evidence for a transparency-efficiency tradeoff in human-machine cooperation. *Nat. Mach. Intell.* **1**, 517–521. 10.1038/s42256-019-0113-5 (2019).

[36] Bröhl, C., Nelles, J., Brandl, C., Mertens, A. & Nitsch, V. Human-robot collaboration acceptance model: Development and comparison for Germany, Japan, China and the USA. *Int. J. Social Robot.* **11**, 709–726. 10.1007/s12369-019-00593-0 (2019).

[37] Persson, A., Laaksoharju, M. & Koga, H. We mostly think alike: Individual differences in attitude towards AI in Sweden and Japan. *Rev. Socionetw. Strategies* **15**, 123–142. 10.1007/s12626-021-00071-y (2021).

[38] Schniter, E., Shields, T. W. & Sznycer, D. Trust in humans and robots: Economically similar but emotionally different. *J. Econ. Psychol.* **78**, 102253. 10.1016/j.joep.2020.102253 (2020).

[39] Liu, P., Zhai, S. & Li, T. Is it OK to bully automated cars?. *Accident Anal. Prevent.* **173**, 106714. 10.1016/j.aap.2022.106714 (2022). 10.1016/j.aap.2022.106714 35613527

[40] Yamakawa, T., Okano, Y. & Saijo, T. Detecting motives for cooperation in public goods experiments. *Exp. Econ.* **19**, 500–512. 10.1007/s10683-015-9451-2 (2016).

[41] von Schenk, A., Klockmann, V. & Köbis, N. Social preferences towards machines and humans. *Perspect. Psychol. Sci.*10.1177/17456916231194949 (2023). 10.1177/17456916231194949 PMC11720266 37751604

[42] Hou, Y. T. & Jung, M. F. Who is the expert? Reconciling algorithm aversion and algorithm appreciation in AI-supported decision making. *Proc. ACM Human–Computer Interaction* **5**, 1–25. 10.1145/3479864 (2021).

