# Supplementary material for: Human cooperation with artificial agents varies across countries
Source: Sci Rep. 2025 Mar 22;15:10000. doi: 10.1038/s41598-025-92977-8 (PMC11929925; doi:10.1038/s41598-025-92977-8)
Supplement: Supplementary file 1 — Supplementary Information. [file 41598_2025_92977_MOESM1_ESM.pdf]

Human cooperation with artificial agents varies across countries

Jurgis Karpus[*], Risako Shirai, Julia Tovar Verba, Rickmer Schulte, Maximilian Weigert, Bahador Bahrami, Katsumi Watanabe, Ophelia Deroy

[*] Corresponding author: Jurgis Karpus (jurgis.karpus@lmu.de)

Contents

Supplementary Fig. S1. The difference between the odds of cooperating with an AI agent and the odds of cooperating with a human is less pronounced in Japan than it is in the United States.

Supplementary Fig. S2. Among participants who predict that their AI co-player will cooperate in the Prisoner's Dilemma game, those in the United States are more confident in their prediction than those in Japan.

Supplementary Fig. S3. People in Japan, like people in the United States, report "extreme" levels of emotion.

Supplementary Fig. S4. The difference between the odds of feeling worse about exploiting a co-player in Japan and the odds of feeling worse about exploiting a co-player in the United States is more pronounced in people's interactions with AI agents than in their interactions with fellow humans.

Supplementary Fig. S5. Demographic analysis of participants' decision to cooperate.

Supplementary Fig. S6. Demographic characteristics of compared samples from Japan and the United States.

Supplementary Fig. S7. Chronological structure of the experiment procedure.

Supplementary figures

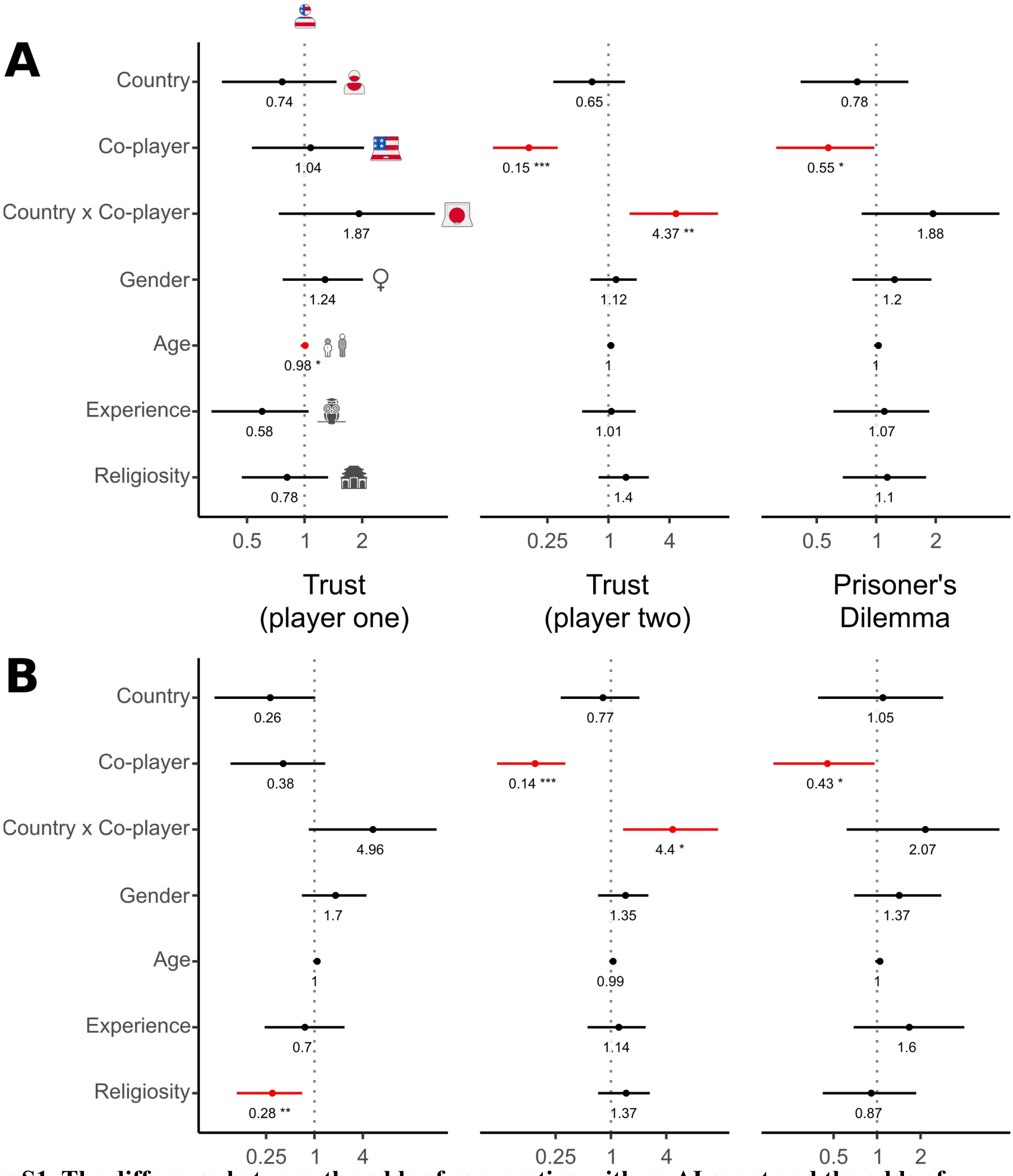

Figure S1. The difference between the odds of cooperating with an AI agent and the odds of cooperating with a human is less pronounced in Japan than it is in the United States. A, The results from a binomial logistic regression with a participant's decision to cooperate as the dependent variable and a participant's country, type of co-player, interaction between the two, gender, age, experience with game theory and/or economics disciplines, and religiosity as independent variables. A deviation from the dotted line indicates an effect on the decision to cooperate. A shift to the right indicates an increase in cooperation (compared to the baseline) among participants in Japan (country), participants who faced an AI agent (co-player), participants who were women (gender), older (age), experienced with game theory and/or economics disciplines (experience), and religious (religiosity). Baseline: men, inexperienced, non-religious participants

in the United States who faced a human co-player. Bars: 95% confidence intervals. Statistically significant effects are highlighted in red: *, **, ***: $p < 0.05$, $p < 0.01$, $p < 0.001$. Note the use of different scales on the x axis for better visualization of results. **B,** The same as above, but limited to the set of participants who predicted that their co-player would cooperate.

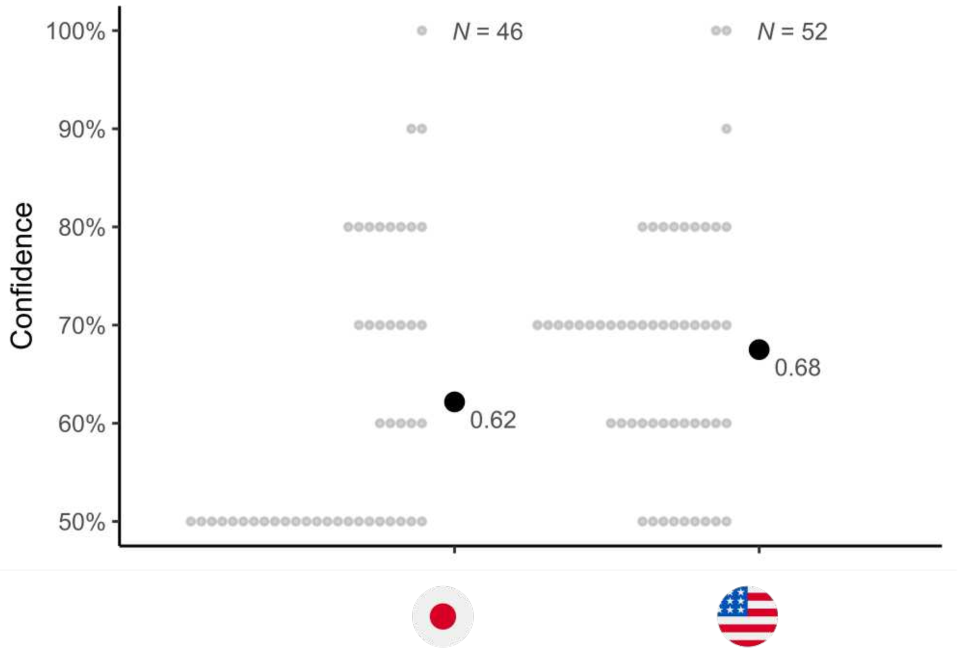

Figure S2. Among participants who predict that their AI co-player will cooperate in the Prisoner's Dilemma game, those in the United States are more confident in their prediction than those in Japan. Participants' reported levels of confidence in their prediction that their AI co-player will cooperate in the Prisoner's Dilemma game, ranging from 50% ("not at all, this is a random guess") to 100% ("very confident, certain"). Grey dots: individual data points. Black dots: means. *N*: the total number of observed confidence ratings (one rating per participant) in Japan and the United States.

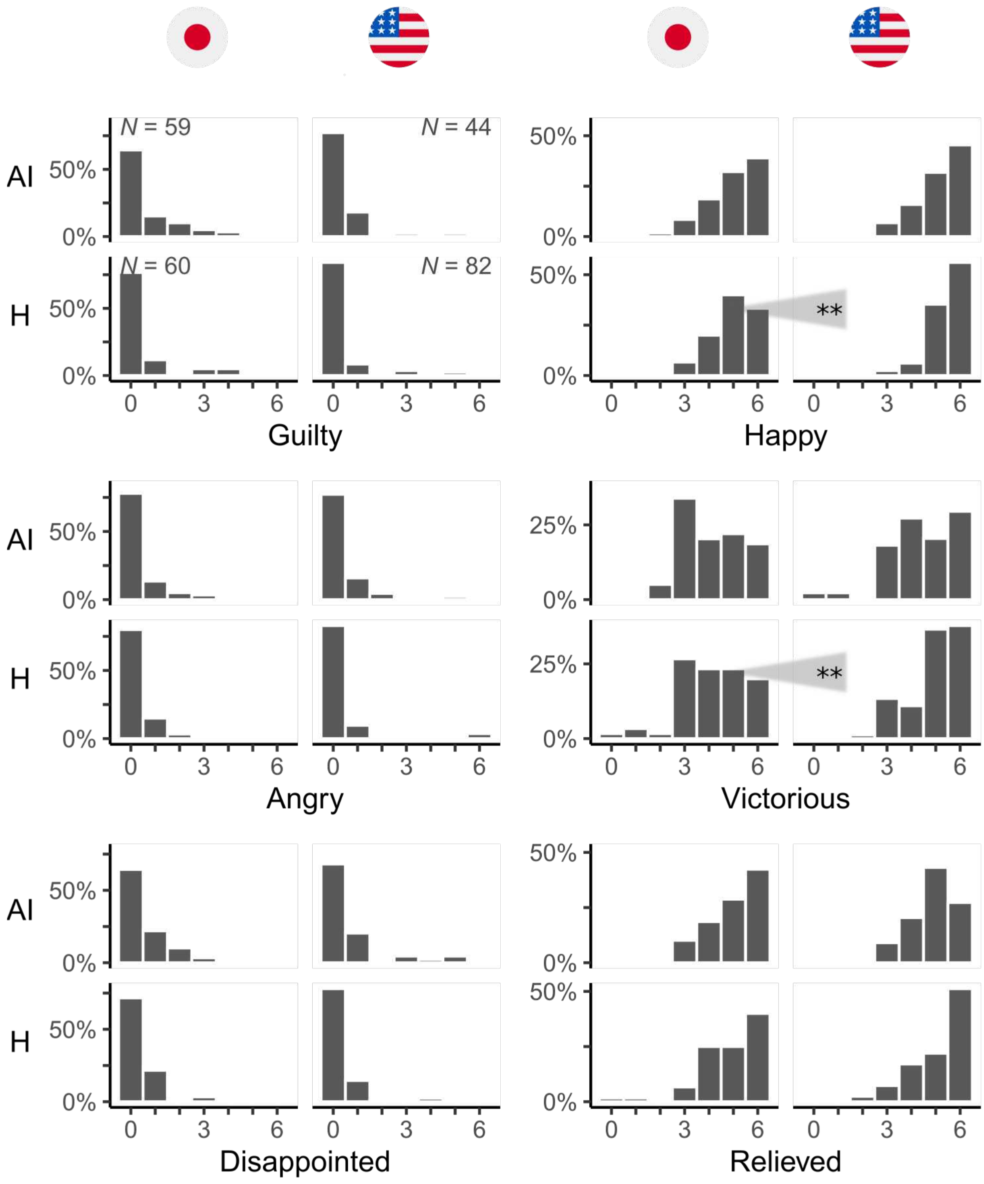

Figure S3. People in Japan, like people in the United States, report "extreme" levels of emotion. The relative frequencies of participants' reported levels of emotion—guilt, anger, disappointment, happiness, victoriousness, and relief—concerning the outcome of a game that they achieved, as measured by a 7-level Likert scale ranging from 0 ("not at all") to 6 ("very"). The results shown are for participants who cooperated when their co-player cooperated as well in both the Prisoner's Dilemma and the Trust games. The distributions on the left for each emotion are of reported levels of emotion among participants recruited in Japan; the distributions on the right are of reported levels of emotion among participants recruited in the United States. The distributions for interactions with AI and human (H) co-players are top and bottom distributions, respectively. The triangular fans indicate a statistically significant proclivity to report a greater level of emotion: **: $p < 0.01$ in two-sided Wilcoxon-Mann-Whitney tests for difference in reported levels of emotion, adjusted using the sequentially rejective Bonferroni procedure recommended by Holm[31] to account

for multiple testing (namely, one test for each emotion). The number of responses (N) in each treatment, displayed in the top-left panel, is the same for all elicited emotions.

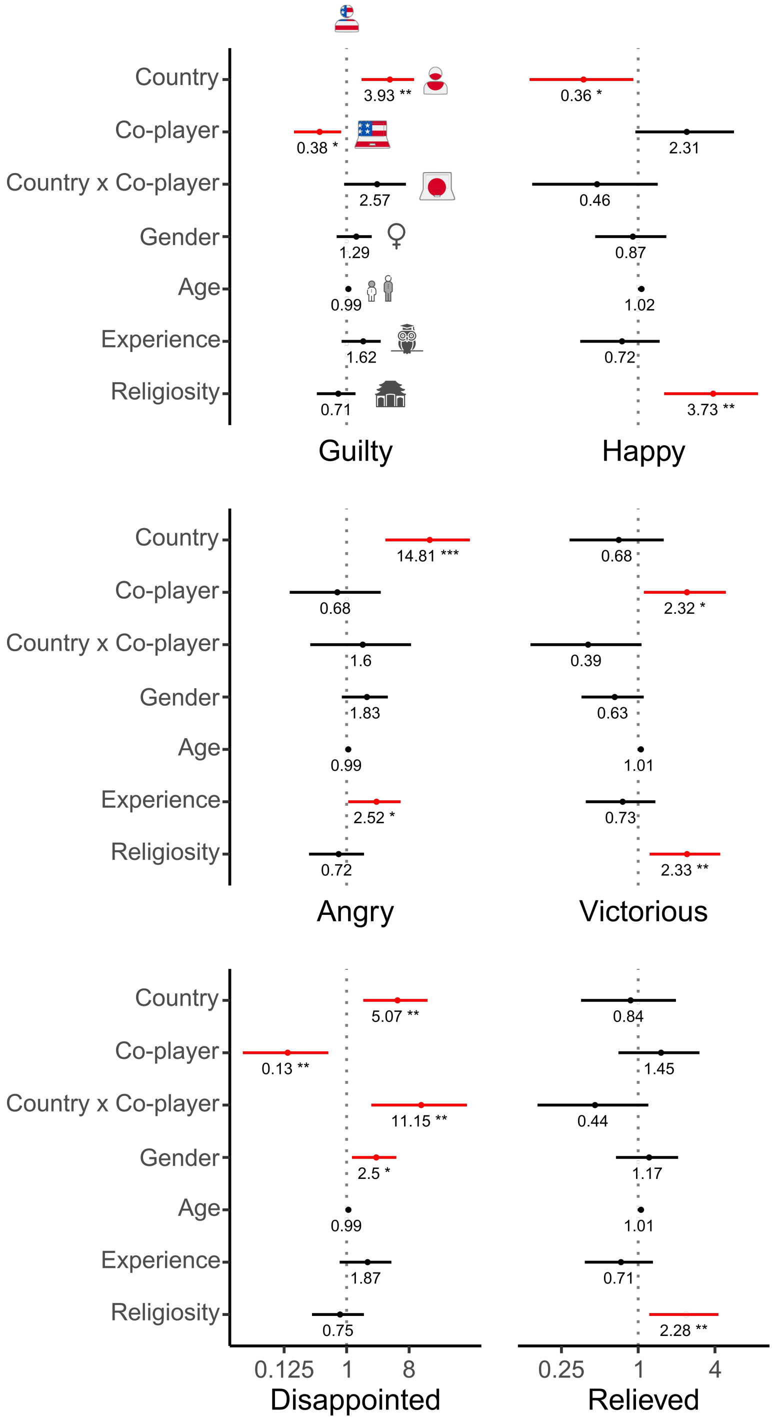

Figure S4. The difference between the odds of feeling worse about exploiting a co-player in Japan and the odds of feeling worse about exploiting a co-player in the United States is more pronounced in people's interactions with AI agents than in their interactions with fellow humans. The results of ordinal logistic regressions with a participant's proclivity to report a higher level of emotion (guilt, anger, disappointment, happiness, victoriousness, and relief) as the dependent variable and a participant's country, type of co-player, interaction between the two, gender, age, experience with game theory and/or economics disciplines, and religiosity as independent variables. The results shown are for participants who exploited

their co-player in a game, which includes those participants who defected in the Prisoner's Dilemma game when their co-player cooperated and those who defected in the Trust game when they played the role of player two. A deviation from the dotted line indicates an effect on the reported level of emotion. A shift to the right suggests a tendency to report a higher level of emotion (compared to the baseline) among participants in Japan (country), participants who faced an AI agent (co-player), participants who were women (gender), older (age), experienced with game theory and/or economics disciplines (experience), and religious (religiosity). Baseline: men, inexperienced, non-religious participants in the United States who faced a human co-player. Bars: 95% confidence intervals. Statistically significant effects are highlighted in red: *, **, ***: $p < 0.05$, $p < 0.01$, $p < 0.001$. Note the use of different scales on the x axis for better visualization of results.

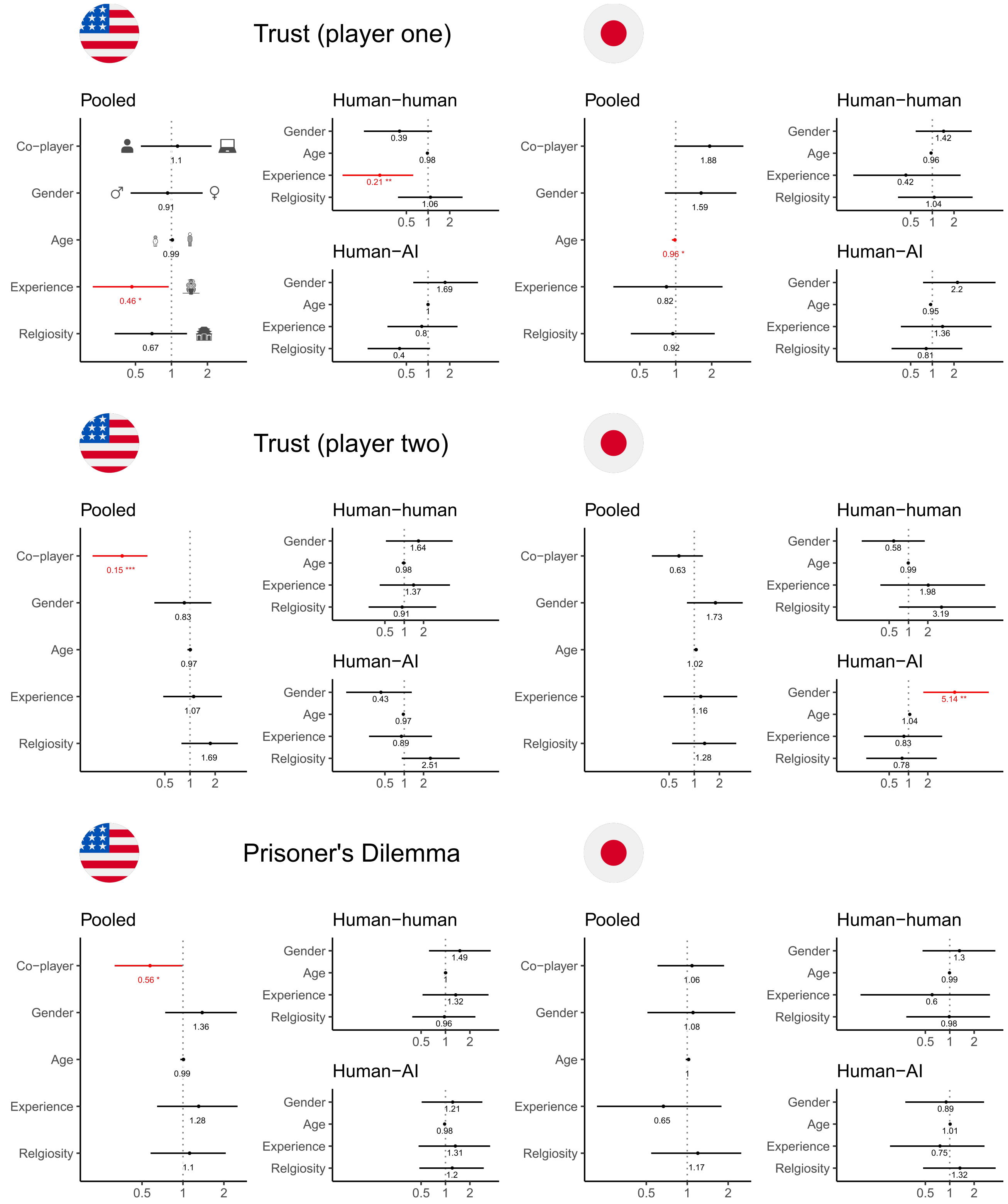

Figure S5. Demographic analysis of participants' decision to cooperate. The results of binomial logistic regressions with a participant's decision to cooperate as the dependent variable and the type of a participant's co-player, participant's gender, age, experience with game theory and/or economics disciplines, and religiosity as independent variables. The plots on the left (right) are for participants who were recruited in the United States (Japan). Pooled: human-human and human-AI treatments combined, where one of the

explanatory variables is the type of a participant's co-player. A deviation from the dotted line indicates an effect on the decision to cooperate. A shift to the right suggests an increase in cooperation (compared to the baseline) among participants who faced an AI agent (co-player), participants who were women (gender), older (age), experienced with game theory and/or economics disciplines (experience), and religious (religiosity). Baseline: men, inexperienced, non-religious participants who faced a human co-player. Bars: 95% confidence intervals. Statistically significant effects are highlighted in red: *, **, ***: $p < 0.05$, $p < 0.01$, $p < 0.001$.

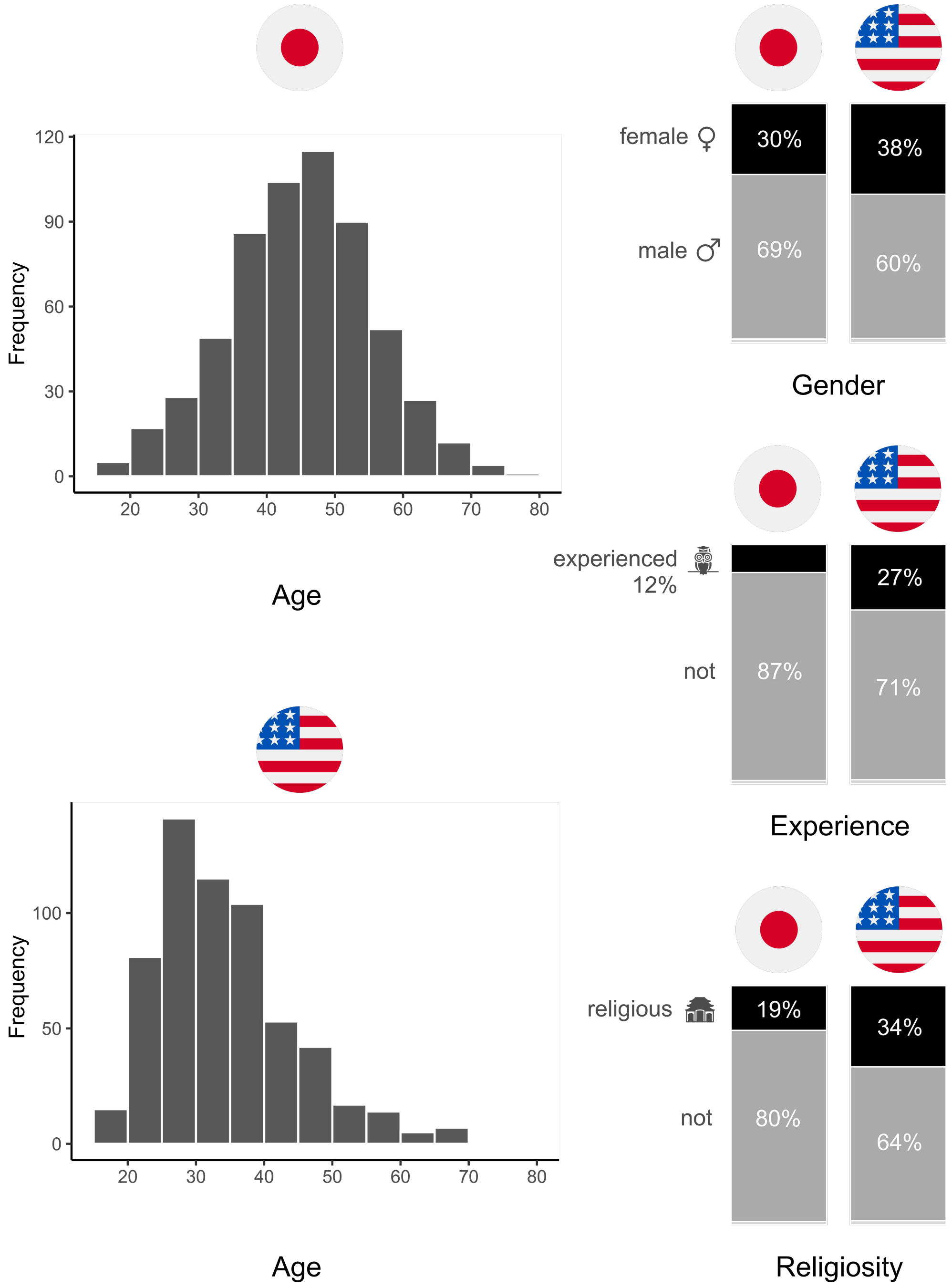

Figure S6. Demographic characteristics of compared samples from Japan and the United States. The percentage values for gender, experience, and religiosity do not add up to 100% within each country due to missing values and/or gender indicated as *other*.

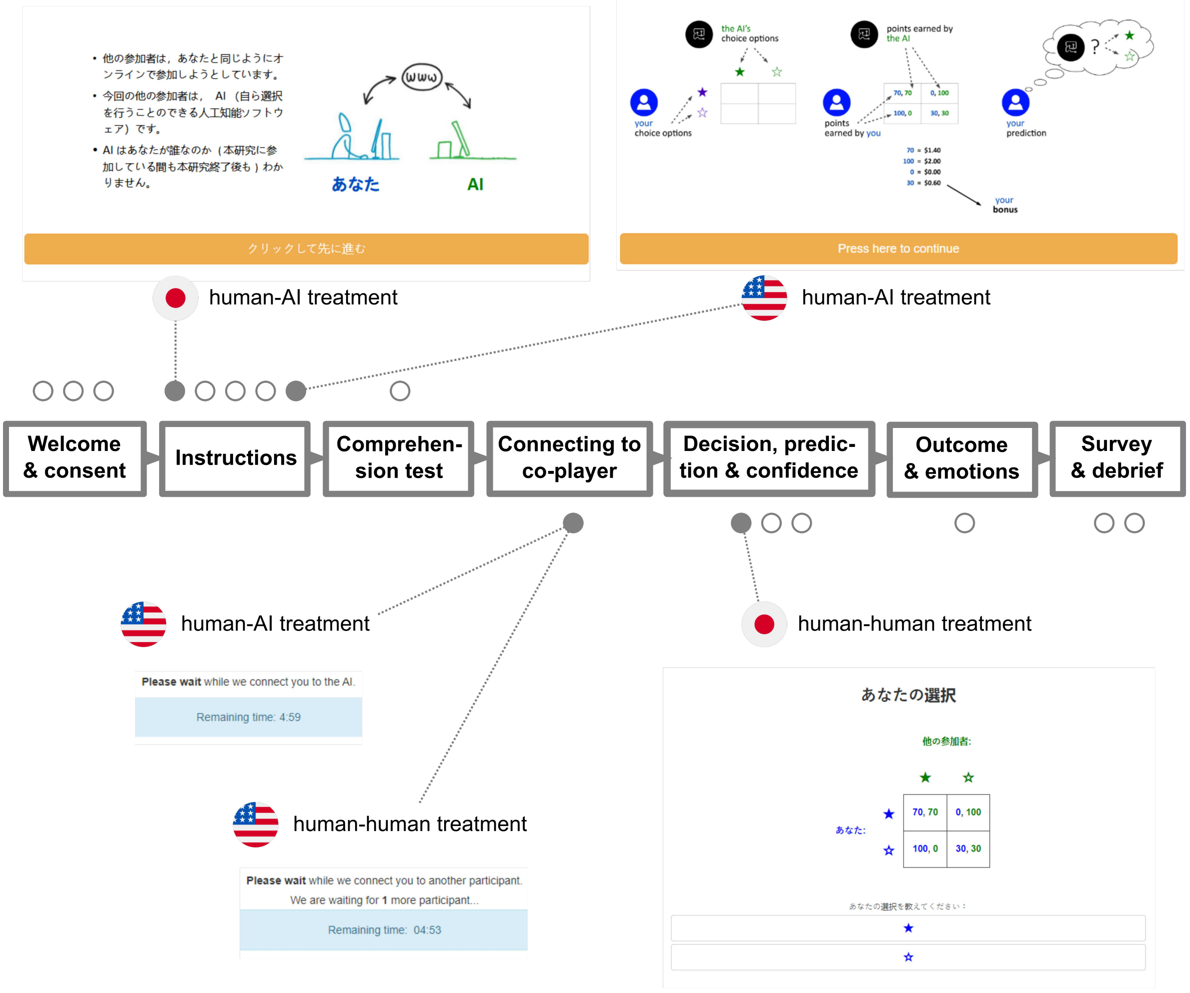

Figure S7. Chronological structure of the experiment procedure. The diagram shows the succession of screens seen and tasks completed by participants with a selection of screenshots. For comparison, some screenshots are from the previously conducted study (in English), in which participants were recruited in the United States.
